# NEDDylation negatively regulates ERRβ expression to promote breast cancer tumorigenesis and progression

**DOI:** 10.1038/s41419-020-02838-7

**Published:** 2020-08-24

**Authors:** Sanoj K. Naik, Eric W.-F. Lam, Monalisa Parija, Surya Prakash, Yannasittha Jiramongkol, Amit K. Adhya, Dilip K. Parida, Sandip K. Mishra

**Affiliations:** 1 Cancer Biology Lab, Gene Function and Regulation Group, Institute of Life Sciences, Nalco square, Chandrasekharpur, Bhubaneswar, Odisha 751023 India; 2 Department of Surgery and Cancer, Imperial Centre for Translational and Experimental Medicine (ICTEM), Hammersmith Hospital, Imperial College London, Du Cane Road, London, W12 0NN UK; 3 Department of Pathology, All India Institute of Medical Sciences, Bhubaneswar, Odisha India; 4 Department of Radiation Oncology, All India Institute of Medical Sciences, Bhubaneswar, Odisha India

**Keywords:** Breast cancer, Breast cancer, Breast cancer, Breast cancer, Preclinical research

## Abstract

Estrogen-related receptor beta (ERRβ) is downregulated in breast cancer cells and its overexpression in breast cancer patients is positively correlated with an improved prognosis and prolonged relapse-free survival. Here, we unravelled a molecular mechanism for ERRβ downregulation in breast cancer. We found that ERRβ is a key substrate of the SCF complex and that NEDDylation can activate the Cullin subunits of the SCF complex to target ERRβ for degradation in breast cancer. Consistently, using in vitro and in vivo models, we demonstrated that MLN4924, a specific small molecule inhibitor of NEDDylation, can restore ERRβ expression and culminate in a reduction in cell proliferation and migration of breast cancer cells. We also showed that increased ERRβ expression promotes the upregulation of its target genes, including the tumour suppressors p21^Cip1/Waf1^ and E-cadherin, involved in cell proliferation and migration arrest at the gene promoter level. Interestingly, this tumour suppressive role of ERRβ does not depend on the expression of ERα in breast cancer. Moreover, our data revealed that the ERRβ recruits the transcription co-activator p300 to its targeted gene promoters to upregulate their expression. Collectively, our work revealed that restoration of ERRβ expression using the NEDDylation inhibitor MLN4924 can be a novel and effective strategy for breast cancer treatment.

## Introduction

Breast cancer is the predominant cause of cancer deaths in underdeveloped countries, representing 14.3% of all cancer deaths. For patients with advanced breast cancer that developed acquired drug resistance and/or disease recurrence or metastasis following first-line chemotherapy, their therapeutic options are very limited^1^. Estrogen has long been known to play a significant role in the development of breast cancer. It is believed that the direct estrogenic effect is mediated by estrogen receptors (ERs), ERα and ERβ, by regulating the expression of their target genes^1^. Estrogen-related receptors (ERRs)^2,3^ share high levels of homology with ERs in their protein structures and are capable of inducing transcription of the estrogen-inducible genes via the ERE and ERRE elements in the promoter region^4,5^. Besides this, estrogen-related receptor beta (ERRβ) has also been proposed to have multiple anti-proliferative properties in breast cancer cells^6^.

NEDDylation is the process of conjugating an ubiquitin-like molecule, NEDD8 (neuronal precursor cell-expressed developmentally downregulated protein 8) to target proteins via a three-step enzymatic reaction, catalysed sequentially by NEDD8-activating enzyme E1 (NAE), NEDD8-conjugating enzyme E2s (UBC12/UBE2M or UBE2F) and substrate-specific NEDD8-E3 ligases^7^. Hitherto, the best-characterised physiological NEDDylation substrates are Cullin family members (Cul-1, 2, 3, 4A, 4B, and 5, other two cullins, Cullin-7 and -9 are less studied), which are scaffold subunits of Cullin–RING ligases (CRLs)^8^. CRLs are the largest family of multiunit E3 ubiquitin ligases, which upon activation by NEDDylation ubiquitinates about 20% of all cellular proteins for targeted degradation via ubiquitin–proteasome system (UPS)^8^.

MLN4924, also known as pevonedistat, is a specific inhibitor of NAE, the E1 enzyme of NEDDylation pathway^9^. Pevonedistat is currently in phase I clinical trial for the treatment of several solid tumours and haematological malignancies^10^. The underlying mechanism of action of MLN4924 is effectively blocking the NEDDylation of CRL complex, resulting in the accumulation of different CRL substrates^11^. Here, we investigated the role of NEDDylation and its impact on ERRβ expression in breast cancer. Furthermore, we explored the molecular mechanism by which ERRβ induces cell cycle arrest and migration inhibition.

## Materials and methods

### Cell culture

Breast cancer cell lines MCF7, T47D and MDA-MB-231 were purchased from National Centre for Cell Sciences (NCCS, Pune, India). MCF7 cells were cultured in Dulbecco′s Modified Eagle′s Medium (DMEM), whereas T47D and MDA-MB-231 cells in RPMI supplemented with 10% fetal bovine serum (FBS) and penicillin–streptomycin (MP Biomedicals, Bengaluru, India) at 37 °C, 5% CO_2_ and 95% humidity. MCF10A, a kind gift from Dr. Annapoorni Rangarajan (IISC, Bangalore, India), was maintained in DMEM F12 containing horse serum supplemented with hydrocortisone, EGF, insulin, cholera toxin and penicillin–streptomycin at 37 °C, 5% CO2 and 95% humidity. The cells were grown until 70–80% confluence and subcultured using Trypsin-EDTA.

### Chemicals

MLN4924 (pevonedistat) was purchased from Merck (5054770001; Bengaluru, India). The stock solution of MLN4924 was prepared in dimethyl sulfoxide (DMSO). The final DMSO concentration for MLN4924 treatment was maintained below 0.01% in the culture medium.

### Cell viability assay

The effect of MLN4924 on viability of cells was examined by 3-(4,5-Dimethylthiazol-2-yl)-2,5 Diphenyltetrazolium Bromide (MTT) assay (ThermoFisher Scientific, Bengaluru, India) (see also Supplementary Materials andMethods).

### Colony forming assay

For colony forming assay, 0.6 × 10^3^ of MCF-7 and MDA-MB-231 cells were seeded in triplicates in 60 mm-plates (Corning, Pune, India) and after 24 h of cell attachment, the cells were treated with 1 μM of MLN4924. (See also Supplementary Materials andMethods).

### Transwell-migration and invasion assay

Transwell-migration assay was performed following manufacturer′s protocol (BD Falcon, Bhubaneswar, India). (See also Supplementary Materials andMethods).

*Western blot analysis*: For western blot, whole-cell lysate of cells were prepared using RIPA buffer [20 mM Tris-HCl (pH 7.5), 150 mM NaCl, 1 mM Na_2_EDTA, 1 mM EGTA, 1% NP-40, 1% sodium deoxycholate, 2.5 mM sodium pyrophosphate, 1 mM β-glycerophosphate, 1 mM Na3VO4 and 1 μg/mL leupeptin] as previously described^12^. The lysed samples were collected after centrifugation for 15 min at 12,000 × *g*, 4 °C. Equal amount (30 μg) of proteins were loaded after Bradford method of protein quantification. The samples were run in 10% SDS-PAGE gel, transferred on PVDF membrane (Millipore) and blocked with 5% (w/v) non-fat milk (Sigma, St Louis, MO, USA). Blots were then incubated with primary antibody overnight [ERRβ (1:5000) (Sc-68879) (Santa Cruz, CA, USA), NEDD8 (1:5000) (GTX54567) (Zeeland, Michigan, United States), App-Bp1 (1:5000) (PAC219Hu01 Cloud-Clone Corp. USA), UBA3 (1:5000) (HPA034873) (Sigma, St Louis, MO, USA), GAPDH (1:10000) (sc-365062) (Santa Cruz, CA, USA), p21^CIP1^ (1:5000) (CST-99323) (Europe, B.V), α-tubulin (1:1000) (T9026) (Sigma St Louis, MO, USA), E-Cadherin (1:5000) (CST-9782T) (Europe, B.V), p300 (1:5000) (P2859) (Sigma St Louis, MO, USA), Thereafter, 1 h with their respective HRP conjugated secondary antibody [anti-rabbit (1:5000, Sigma Aldrich) or anti-mouse (1:5000, Sigma Aldrich)], the blots were subjected to chemi-luminescent detection reagent for visualisation and the bands were detected by using Gel Doc™ XR + Imager. Densitometry analyses of the protein bands were calculated by using ImageJ software.

*Transfection and luciferase assays*: MCF7 cells were grown in 24 well-plates in phenol red free DMEM supplemented with 10% (v/v) charcoal treated FBS, 24 h prior to estrogen (E2) treatment. Cells were transfected with, pEYFP C1-ERRβ, pGL2-P21, pGL3-E-Cadherin, and pRL-Renilla luciferase constructs (Promega Biotech, Bhubaneswar, India) in different combinations using jetPRIME-polyplus-transfection reagent (Polyplus transfection, New York, NY, USA) according to manufacturer′s protocol. Luciferase assay was performed using Dual luciferase assay detection kit (Promega) according to manufacturer′s protocol. Luciferase readings were obtained and were normalised with Renilla luciferase activity. The graph was plotted with normalised readings using GraphPad Prism software version 6.01.

*Gene silencing with small interfering RNAs (siRNAs)*: To perform gene silencing, Culin-1 (5′-GGUUAUAUCAGUUGUCUAA-3′) and non-targeting control siRNAs (Eurogentec, Seraing, Belgium) were transfected into the cells using INTERFERin® PolyPlus siRNA transfection reagent (Polyplus transfection, France; Ref# 409−10) according to the manufacturer′s instructions.

*Tissue microarray*: Breast cancer tissue microarray slides (Cat No. BR 246a) were purchased from US Biomax (Rockville, MD, USA). The slides were stained by anti-APP-BP1(PAC219Hu01 Cloud-Clone Corp. USA) and anti-NEDD8 (GTX54567) (Zeeland, Michigan, United States) antibody at 1:50 dilution and were further processed using ABC system (Vector Laboratories, Bulingame, CA, USA) as described previously^13^. (See also Supplementary Materials andMethods).

*Chromatin immunoprecipitation assay (ChIP)*: Chromatin immunoprecipitation was performed as previously described with minor modifications^12^. (See also Supplementary Materials andMethods).

*Co-immunoprecipitation*: Cells were washed with PBS pH 7.4 twice and lysed with NP40 buffer (50 mM Tris-Cl pH 8.0, 150 mM NaCl, 1% NP40). Lysates were precleared by the addition of 50 μl of agarose beads for 30 min. Total protein (600 μg) and 4 μg of antibody were used for each IP and rotated overnight in 4 °C. Beads (30 μg) were added to each IP and rotated for 2 h, followed by centrifugation at 500 × *g* for 3 min. Supernatants were removed, and pellets were washed four times with NP40 buffer. Complexes were eluted in SDS lysis buffer.

## Results

### ERRβ protein expression is downregulated in breast cancer

**Fig. 1:**
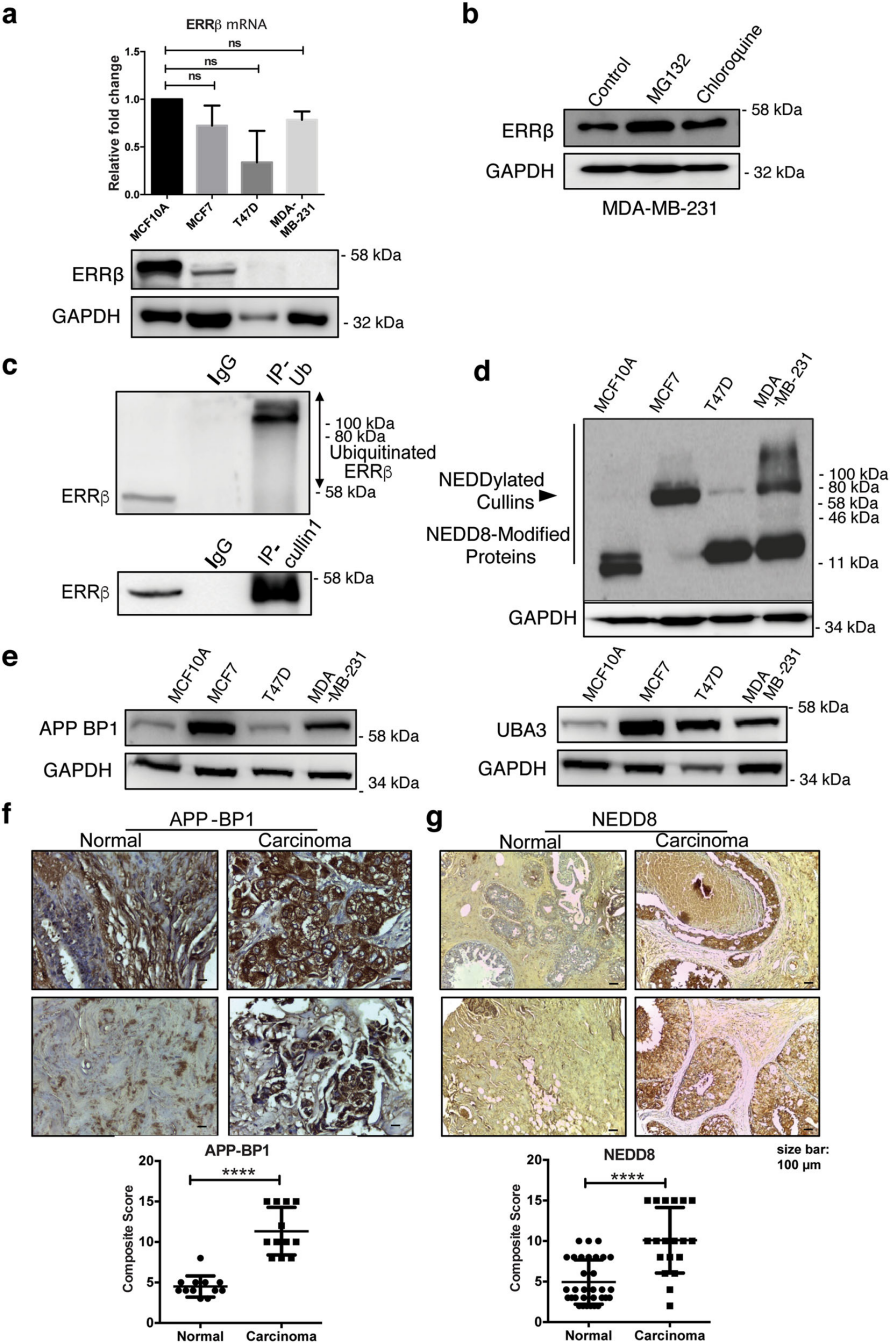
ERRβ protein expression is downregulated by NEDD8 activity in breast cancer. **a** Relative ERRβ mRNA expression level of MCF10A, MCF7, T47D and MDA-MB-231 cells were analysed using qRT-PCR. Three technical repeats (*N* = 3) were performed and the data represent the means ± SD. (two-tailed *t* test; Significant: ns no significant difference) (upper panel). Representative western blot analysis of the ERRβ in MCF10A, MCF7, T47D and MDA-MB-231 cells. GAPDH was used as a loading control (lower panel). **b** MDA-MB-231 cells were treated with 1 µM MG132 and Chloroquine independently for 12 h and the ERRβ protein expression was analysed by western blotting. Representative Western blot is shown. GAPDH was used as a loading control. (*N* = 3). **c** Co-immunoprecipitation was performed with antibodies against Ubiquitin (Ub) and Cullin1 and analysed with ERRβ antibodies using western blotting. Anti-IgG antibody was used as a negative control. **d** Western blot analysis of the NEDD8 in in MCF7, MCF10A, T47D and MDA-MB-231 cells. GAPDH was used as a loading control. (*N* = 3). **e** APP-BP1 and UBA3 expression was analysed by western blotting in MCF10A, MCF7, T47D and MDA-MB-231 cells. GAPDH was used as a loading control. (*N* = 3). **f**, **g** Immunohistochemical (IHC) staining of normal (left) and carcinoma (right) breast tissue samples using the APP-BP1 and NEDD8 antibody, respectively. Graphical representation (below) was the IHC composite score of each tissue microarray sample. A composite score (<3 = low; 3–5 = moderately; ≥6 = highly categorised) was calculated for each sample using intensity score and percentage of APP-BP1 and and NEDD8 stained-positive cells. (2-sample *t* test; significant: ****p < 0.001, very significant). Bar = 100 μm.

Previously, we have reported the expression levels of ERRβ are lower in breast cancer patients as well as in cell lines compared to their normal counterparts^14^. To explore the mechanism associated with ERRβ downregulation, we first analyzed the transcript levels of ERRβ in a non-tumorigenic epithelial cell line (MCF10A), two ER-positive (MCF7 and T47D) and a triple-negative (MDA-MB-231) breast cancer cell lines. Quantitative RT-PCR analysis revealed insignificant differences in ERRβ transcript levels between these cell lines, but western blot analysis showed that ERRβ is substantially downregulated at the protein level in the three breast cancer cell lines when compared with the non-cancerous MCF10A breast epithelial line (Fig. 1a). At the post-transcriptional level, protein degradation is primarily mediated through an ubiquitin–proteasome- or a lysosomal-dependent pathway, which can be responsible for the downregulation of ERRβ in breast cancer cells^15^. To test this conjecture, we treated the MDA-MB-231 with 1 µM MG132 and chloroquine independently and determined the expression of ERRβ by western blotting. An increase in ERRβ expression was evident in cells treated with the proteasome inhibitor MG132 but not in cells cultured with the lysosomal inhibitor chloroquine (Fig. 1b), suggesting that ERRβ expression is modulated by protein degradation mediated by the ubiquitin–proteasome pathway. To further confirm the downregulation of ERRβ is predominantly at the protein but not the transcript level, we analysed the ERRβ mRNA levels in a number of cancer microarrays in ONCOMINE^16^. The mRNA levels of ERRβ from breast cancer patients were not significantly different from the normal controls in most microarrays studied (significance: n.s. *P* > 0.05, respectively) (Supplementary Fig. 1). However, analysis of the TGCA breast samples from normal and cancer patients showed that there is a small but significant difference in ERRβ mRNA levels between the two groups (Significant: *P < 0.05)^17^ (Supplementary Fig. 1). Altogether, these data suggested that ERRβ is at least partially downregulated at the protein level by the ubiquitin–proteasome pathway in breast cancer.

### NEDD8 and NEDD8-activating enzymes are overexpressed and positively correlated with breast cancer poor prognosis

To study the molecular mechanism of ERRβ downregulation at the protein level, we first sought to identify the regulator of ERRβ degradation. Co-immunoprecipitation assay revealed an interaction of ERRβ with ubiquitin (UB) and Cullin1 (Fig. 1c). As Cullin1 is a component of SCF complex which belongs to a large family of E3 ubiquitin ligases^18^, this result suggested that the Skp, Cullin, F-box containing (SCF) complex may play a vital role in the downregulation of ERRβ in breast cancer. NEDDylation of Cullin1 has been shown to enhance the ubiquitination activity of the SCF complex and promote target protein turnover^19^. To investigate the status of NEDDylation pathway in breast cancer, we first determined the expression levels of NEDD8 in the MCF7, T47D, MDA-MB-231 and MCF10A cells. The result showed that NEDD8 was overexpressed in the breast cancer cell lines tested compared to the non-cancerous breast epithelial MCF10A cells (Fig. 1d).

We further investigated the expression of NEDD8-activating enzyme (NAE), a heterodimer of APP-BP1 and UBA3, which determines the activity of NEDD8 and the NEDDylation pathway. Western blot analysis showed that the expression levels of both NAE enzymes were significantly higher in the breast cancer cells (Fig. 1e), with the highest expression of APP-BP1 and UBA3 observed in the estrogen receptor alpha (ERα) positive MCF7 cells (Fig. 1e). As APP-BP1 catalyses the rate-limiting step of NEDDylation^20^, we, next, determined APP-BP1 and NEDD8 expression in tissue microarrays from breast cancer patients and the result revealed that both APP-BP1 and NEDD8 are upregulated in breast cancer (Fig. 1f, g), suggesting breast cancer cells exhibit higher levels of NEDDylation than normal cells. In concordance with a tumour suppressive role for NEDD8, Kaplan–Meier analysis using the K–M plotter^21^ also showed a poorer disease-free survival rate in patients, significantly associated with elevated UBA3, APP-BP1 and NEDD8 mRNA expression (*P* = 4.0 × 10^−5^, 2.0 × 10^−13^ and 2.2 × 10^−5^, respectively) (Supplementary Fig. S2). Taken together, these data proposed that an elevated NEDDylation in breast cancer promotes ERRβ downregulation through ubiquitination mediated by the Cullin1-containing SCF complex.

### Inhibition of NEDDylation by MLN4924 restores ERRβ expression in breast cancer in vitro and in vivo

**Fig. 2:**
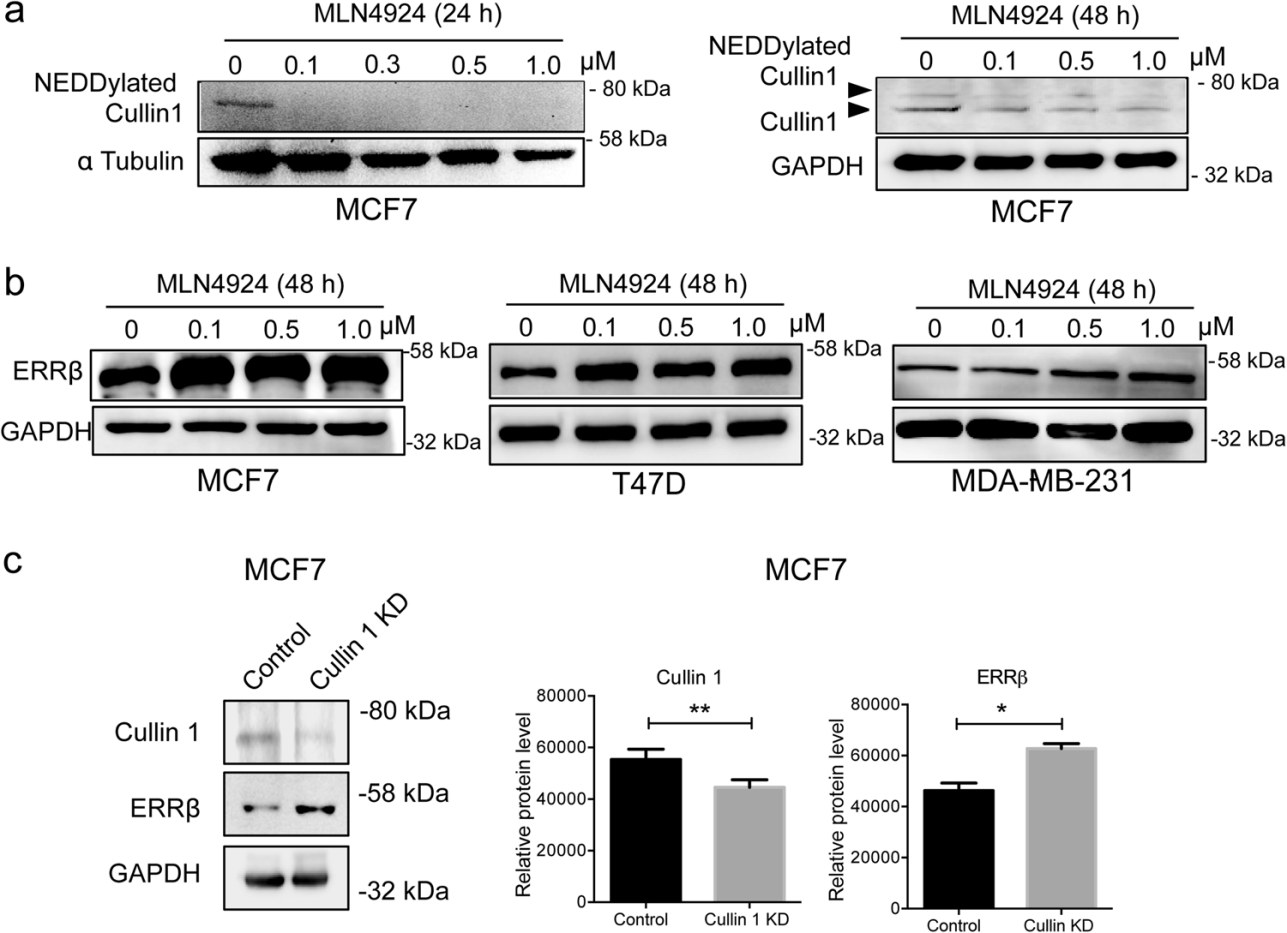
ERRβ expression is regulated by NEDDylation and the SCF complex complex in breast cancer. **a** MCF7 cells were treated with varying concentrations of MLN4924 (0, 0.1, 0.3, 0.5 and 1.0 µM) for 24 h prior to western blot analysis of NEDDylated Cullin1. The NEDD8-modified Cullin1 was determined by probing the whole western blot membrane with an anti-NEDD8 antibody. The characteristic predominant band (just below 80kD) correspond to the NEDD8-modified Cullin1, were shown. Tubulin was used as a loading control (left panel). MCF7 cells were treated with varying concentrations of MLN4924 (0, 0.1, 0.3, 0.5 and 1.0 µM) for 48 h prior to western blot analysis of hyper-NEDDylated and hypo-NEDDylated Cullin1. GAPDH was used as a loading control (right panel). **b** MCF7, T47D and MDA-MB-231 cells were treated with varying concentrations of MLN4924 (0, 0.1, 0.5 and 1.0 µM) for 48 h prior to western blot analysis of ERRβ. GAPDH was used as a loading control. (*N* = 3). **c** Expression levels of ERRβ and Cullin1 in MCF-7 cells were analysed by western blotting after transfection with siRNA targeting Cullin1 or non-targeting control siRNA. Western blots are representative of three independent experiments. The ratio to GAPDH expression was calculated for the relative ERRβ and Cullin1 expression levels. The relative expression levels (right panels) are shown. Data represent means ± SD. (*n* = 3; *t* tests). Significant *P < 0.05; **P < 0.01.

NEDDylation of the Cullin subunit promotes the assembly of the SCF complex and the induction of ubiquitin ligase activity^20^. To test if NEDDylation is involved in the downregulation of ERRβ expression, we first treated the MCF7 cells with MLN4924 (pevonedistat), a selective small molecule inhibitor of NAE^22^, for 24 h to investigate its effect on Cullin1 NEDDylation^20^. Cullin1 is the major NEDDylation target in MCF7 cells. The characteristic predominant band (just below 80 kD) correspond to the NEDD8-modified Cullin1 was revealed after probing with an anti-NEDD8 antibody (Fig. 2a; left panel). The result showed that Cullin1 NEDDylation decreased after MLN4924 treatment in a dose-dependent manner. To confirm this, MCF7 cells were treated with varying concentrations of MLN4924 for 48 h prior to western blotting for hyper-NEDDylated and hypo-NEDDylated Cullin1 with an anti-Cullin1 antibody. The result showed that the slower migrating hyper-NEDDylated Cullin1 species decreased with MLN4924 treatment (Fig. 2a; right panel). Together, the results showed that MLN4924 reduced the levels of NEDDylated Cullin1 in a dose-dependant manner, confirming that Cullin1 are NEDDylated in MCF7 cells (Fig. 2a).

To confirm that elevated Cullin1 NEDDylation downregulates ERRβ expression in breast cancer, we treated different breast cancer cell lines (MCF7, T47D and MDA-MB-231) with varying concentrations of MLN4924 (0, 0.1, 0.3, 0.5 and 1.0 μM) for 48 h and determined the expression of ERRβ by western blotting. Here, the expression of ERRβ was upregulated by MLN4924 in a dose-dependent manner (Fig. 2b). To further confirm that ERRβ is a substrate of the SCF complex, we examined the effects of knockdown of Cullin1, an essential component of the SCF complex. The results showed that Cullin1 depletion caused an upregulation in ERRβ expression (Fig. 2c). Together the data suggested that NEDDylation promotes the ubiquitylation activity of SCF complex to limit ERRβ expression in breast cancer.

### MLN4924 reduces ERRβ ubiquitination to protect it from proteasomal-mediated degradation

**Fig. 3:**
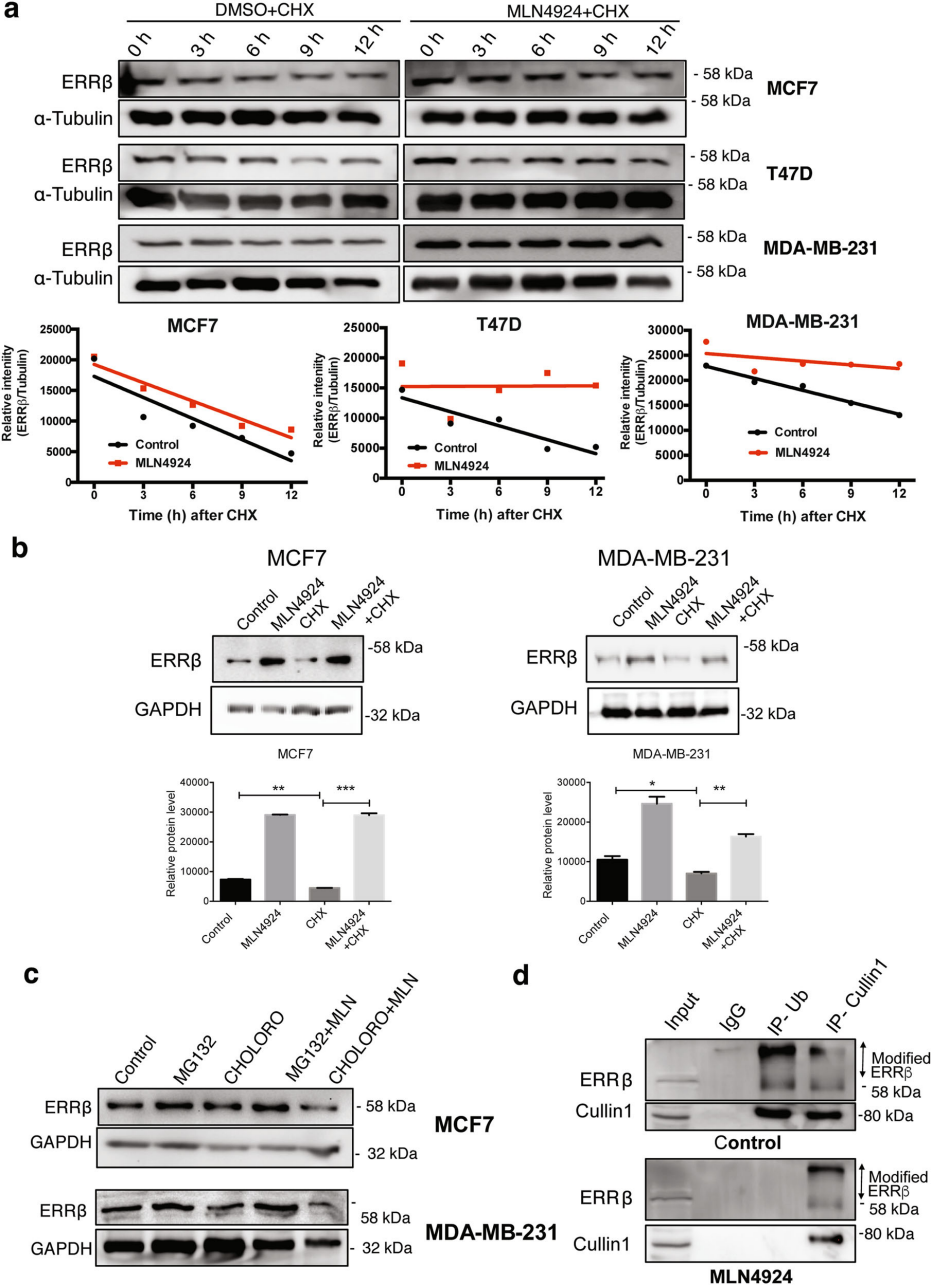
MLN4924 (pevonedistat) restores ERRβ expression in breast cancer through reducing its ubiquitination and proteasomal-mediated degradation. **a** MCF-7, T47D and MDA-MB-231 cells were treated with 25 μg/mL cycloheximide (CHX) in the presence of 0.3 µM MLN4924 or DMSO as a control. Cells were harvested in 3 h intervals (0, 3, 6, 9, 12 h) for western blot analysis of ERRβ protein expression. α-Tubulin was used as a loading control. Densitometry was used to quantify the ERRβ and α-Tubulin levels from which independent background readings were subtracted. Western blots are representative of three independent experiments. The relative expression levels (lower panels) are shown. **b** MCF7 and MDA-MB-231 cells were treated with 25 μg/mL cycloheximide (CHX) in the presence of 0.3 µM MLN4924 or DMSO as a control. Cells were harvested after 24 h for western blot analysis of ERRβ protein expression. GAPDH was used as a loading control. Densitometry was used to quantify the ERRβ and GAPDH levels from which independent background readings were subtracted. Western blots are representative of three independent experiments. The relative expression levels (lower panels) are shown. Data represent means ± SD. (*n* = 3; *t* tests). Significant *P < 0.05; **P < 0.01; ***P < 0.001. **c** Western blot analysis of ERRβ expression in different combinations of MLN4924, MG132 and chloroquine-treated MCF7 (left) and MDA-MB-231 (right) cells. GAPDH and α-Tubulin were used as a loading control, respectively. (*N* = 3). **d** Co-immunoprecipitation was performed on 0.3 µM MLN4924-treated and control untreated MCF7 cells (24 h) with antibodies against Ubiquitin (Ub) and Cullin1 and analysed with ERRβ and Cullin1 antibodies using western blotting. Anti-IgG antibody was used as a negative control. (*N* = 3).

We next performed cycloheximide (CHX) experiments to block protein translation and determined the ERRβ turnover rates upon MLN4924 treatment. We found that NEDDylation inactivation by MLN4924 decreased the rates of ERRβ degradation in the breast cancer MCF7, T47D and MDA-MB-231 cell lines (Fig. 3a, b). Next, we tested whether MLN4924 regulates ERRβ expression at the transcriptional level. Indeed, the transcription of ERRβ was not significantly affected by the MLN4924 treatments in both the MCF7 and MDA-MB-231 cells (Supplementary Fig. S3). These results suggested that ERRβ is stabilised upon NEDDylation inactivation by MLN4924 at the post-translational level. We next examined whether ERRβ is downregulated by proteasomal or lysosomal degradation, and treated both MCF7 and MDA-MB-231 cells with the proteasome inhibitor MG132 and the lysosome inhibitor chloroquine in the absence or presence of MLN4924. The results showed that ERRβ expression was upregulated by MG132 alone and also with MG132 and MLN4924 but not with chloroquine alone or with chloroquine and MLN4924 (Fig. 3c). These results further supported our previous findings that MLN4924 inhibits the SCF complex to reduce ERRβ ubiquitination and proteasomal degradation and that, ERRβ is a substrate of SCF complex. To confirm this, a co-immunoprecipitation assay was employed to examine the levels of ERRβ ubiquitination and interaction with Cullin1 in MLN4924-treated and untreated (control) MCF7 cells (Fig. 3d). The results showed that ERRβ bound to Cullin1 but was no longer ubiquitinated in the presence of MLN4924 and confirmed that MLN4924 promotes ERRβ protein accumulation through a repression of the SCF complex activity and, ERRβ ubiquitination and degradation.

### Upregulation of ERRβ expression is required for MLN4924-induced cell growth suppression

**Fig. 4:**
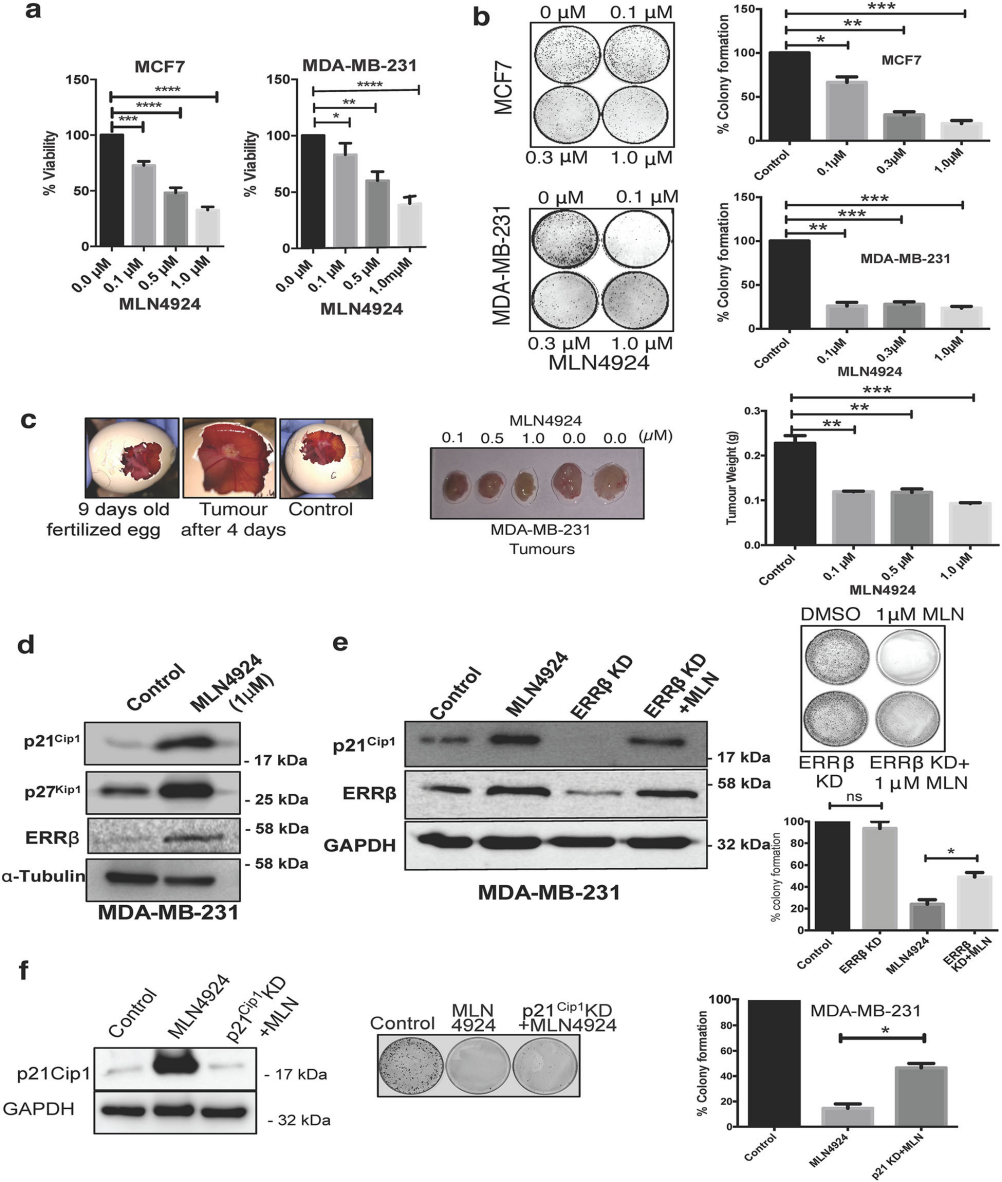
Upregulation of ERRβ expression is required for MLN4924-induced cell growth suppression. **a** MCF-7 and MDA-MB-231 cells were treated with varying concentrations of MLN4924 (0, 0.1, 0.5, 1.0 μM) for 48 h and analyse for the cell proliferation rates with MTT assay. Graphical representation was the optical density (wavelength = 570 nM) of the MLN4924-treated MCF-7 and MDA-MB-231 cells relative to the untreated control counterparts. (*N* = 3). **b** MCF-7 and MDA-MB-231 cells were treated with varying concentrations of MLN4924 (0, 0.1, 0.3 and 1.0 µM) for 10 days. The representative images showed cell clonogenic survival of the MLN4924-treated MCF7 and MDA-MB-231 cells (left panels). Data represent means ± SD. (*n* = 3; *t* tests). Significant *P < 0.05; **P < 0.01; ***P < 0.001. **c** MDA-MB-231 cells were treated with varying concentrations (0, 0.1, 0.5 and 1.0 µM) of MLN4924 and injected onto the chorioallantoic membrane of 9 days old fertilised chick egg xenograft model (left panel). The representative images showed the dissected tumour after a 5 days incubation (central panel). (*N* = 3). Data are representative of three independent experiments. Data represent means ± SD. (*n* = 3; *t* tests). Significant **P < 0.01; ***P < 0.001. **d** MDA-MB-231 cells were treated with 1 µM MLN4924 for 24 h and the protein expression of p21^Waf1/Cip1^ and p27^Kip1^ were analysed by western blotting. α-Tubulin was used as a loading control. (*N* = 3). **e** MDA-MB-231 cells were transfected with ERRβ shRNA and/or treated with 1.0 µM MLN4924. The representative images showed cell clonogenic survival of the MLN4924-treated, with and without ERRβ-knockdown, MDA-MB-231 cells. (*N* = 3) (top right). Data represent means ± SD. (*n* = 3; *t* tests). Significant; *P < 0.05; ns non-significant. (bottom right). **f** MDA-MB-231 cells were treated with 1.0 µM MLN4924 in the presence or absence of p21^Waf1/Cip1^ shRNA-mediated knockdown. The protein expression of p21^Waf1/Cip1^ was analysed by western blotting with or without p21^Waf1/Cip1^ depletion in the presence of 1.0 µM MLN4924. GAPDH was used as a loading control. The representative images showed cell clonogenic survival of the MLN4924-treated, with and without p21^Waf1/Cip1^-knockdown, MDA-MB-231 cells. (*N* = 3) (middle). Data represent means ± SD. (*n* = 3; *t* tests). Significant; *P < 0.05 (right).

A previous report suggested that the downregulation of ERα expression is necessary for the MLN4924-induced cell proliferation suppression^23^. However, the study did not address the effect of MLN4924 in ERα-negative and triple-negative breast cancer cells. To investigate this, MCF7 (ERα-positive) and MDA-MB-231 (triple-negative) breast cancer cell lines were chosen for proliferative and clonogenic survival analysis (Fig. 4a, b). The result revealed that MLN4924 significantly reduced the cell proliferation and clonogenic survival in both MCF7 and MDA-MB-231 cell lines (Fig. 4a, b). We, next, studied the consequence of MLN4924 on the induction of ERRβ expression in vivo using the chick chorioallantoic membrane (CAM) xenograft model. The untreated and MLN4924-treated MDA-MB-231 cells were injected onto the chorioallantoic membrane of 9 days old fertilised chick egg through a window opened on the eggshell. In concordance, the chick CAM xenograft model also showed that MLN4924 inhibits tumour growth in vivo from MDA-MB-231 cells (Fig. 4c), further confirming that MLN4924 has anti-proliferative functions in both ER-positive as well as ER-negative breast cancer cells. To further validate MLN4924 anti-proliferative functions, MCF7 and MDA-MB-231 cells were treated with 1 µM MLN4924 for 24 h and the proteins were extracted for western blot analysis. In agreement with a previous study showing that MLN4924 causes an accumulation of the negative cell cycle regulators p21^Waf1/Cip1^ and p27^Kip1^ to induce proliferative arrest^24^, our western blot data suggested that MLN4924 treatment promotes p21^Waf1/Cip1^ and p27^Kip1^ expression to reduce cell proliferation of MCF7 and MDA-MB-231 cells (Fig. 4d and Supplementary Fig. S4). To determine whether the MLN4924-induced ERRβ expression is required for the proliferative arrest, we silenced ERRβ and evaluated the effect of MLN4924-induced cell growth inhibition. ERRβ depletion caused a downregulation in p21^Waf1/Cip1^ expression (Fig. 4e). The knockdown of ERRβ partially overrode the decrease in clonogenicity induced by MLN4924 in MDA-MB-231 cells (Fig. 4e), suggesting that ERRβ has a role in the anti-proliferative functions of MLN4924. Similarly, we also found that p21^Waf1/Cip1^ knockdown partially overcame the inhibition in clonogenicity by MLN4924 in MDA-MB-231 cells, also suggesting that p21^Waf1/Cip1^ is a key downstream target of the NEDD8 inhibitor MLN4924 (Fig. 4f).

### MLN4924 upregulates p21^Waf1/Cip1^ through ERRβ to inhibit breast cancer growth

**Fig. 5:**
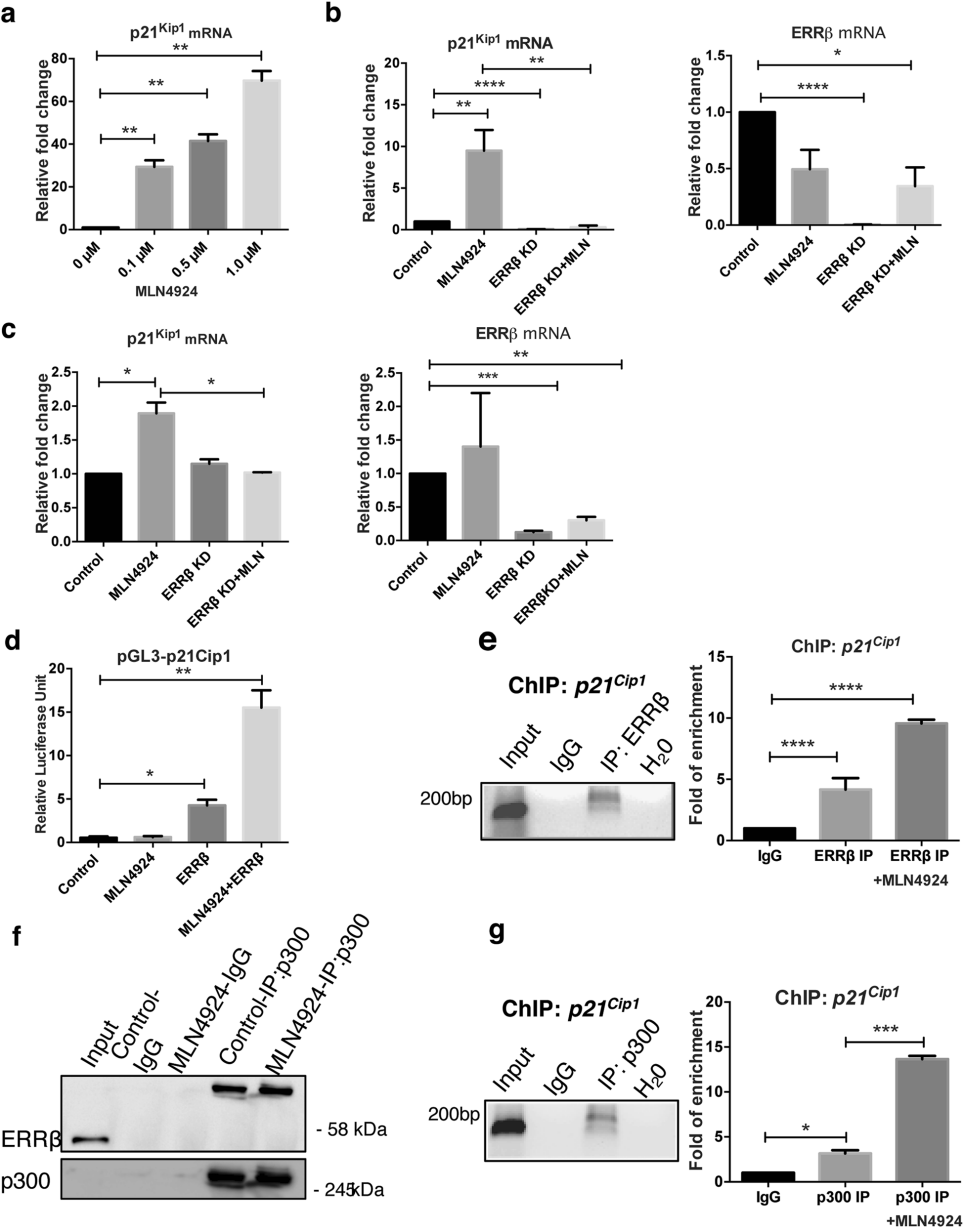
MLN4924 upregulates p21^*Waf1/Cip1*^ through ERRβ to inhibit Breast cancer growth. **a** MCF7 cells were treated varying concentrations of MLN4924 (0, 0.1, 0.5 and 1.0 µM) for 48 h, and relative mRNA expression level of p21^Waf1/Cip1^ were analysed with qRT-PCR. (*N* = 3) The data represent the means ± SD. (two-tailed *t* test; Significant: **P < 0.01, significant) **b** MCF7 and **c** MDA-MB-231 cells were transfected with ERRβ shRNA and relative mRNA levels of p21^Waf1/Cip1^ were analysed after 1 µM MLN4924 treatment for 48 h. (*N* = 3). The data represent the means ± SD. (two-tailed *t* test; Significant: *P < 0.05; **P < 0.01; ***p < 0.001; ****p < 0.0001, significant). **d** MCF7 cells were transfected with p21^Waf1/Cip1^ gene cloned upstream of the pGL2 luciferase reporter and ERRβ shRNA, with and without 1.0 µM MLN4924 treatment. Graphical representation showed relative luciferase unit of the Renilla luciferase reporter activity. (*N* = 3). The data represent the means ± SD (One-way ANOVA test; Significant: *P < 0.05; **P < 0.01, significant). **e** Chromatin Immunoprecipitation was performed with ERRβ antibody on the promoter region of endogenous *p21*^*Waf1/Cip1*^ and analysed with Electrophoresis (left; image colour inverted) and qRT-PCR (right). Anti-IgG antibody was used as a negative control. (*N* = 3). The data represent the means ± SD. (two-tailed *t* test; Significant: ****p < 0.0001, significant). **f** Co-immunoprecipitation was performed with p300 antibody in untreated and 1 µM MLN4924-treated MCF7 cells and analysed with ERRβ and p300 using western blotting. Anti-IgG antibody was used as a negative control. (*N* = 3). **g** Chromatin Immunoprecipitation was performed with p300 antibody on the promoter region of endogenous p21^Waf1/Cip1^ and analysed with electrophoresis (left; image colour inverted) and qRT-PCR (right). Anti-IgG antibody was used as a negative control. (*N* = 3). The data represent the means ± SD. (two-tailed *t* test; Significant: *P < 0.05; ***p < 0.001, significant).

We and others have previously reported that overexpression of ERRβ leads to an upregulation of p21^Waf1/Cip1^ at the transcriptional level^5,14^. We next tested if MLN4924 treatment also modulates p21^Waf1/Cip1^ expression. To investigate this, we treated MCF7 cells with increasing doses (0, 0.1, 0.5 and 1.0 µM) of MLN4924 and determined the mRNA expression level of p21^Waf1/Cip1^ by qRT-PCR. The results revealed p21^Waf1/Cip1^ mRNA levels were induced by MLN4924 in a dose-dependent manner (Fig. 5a). To verify whether ERRβ is essential for the induction of p21^Waf1/Cip1^ expression after MLN4924 treatment, we depleted ERRβ using shRNA vector and, treated MCF7 and MDA-MB-231 cells with 1 µM MLN4924 prior to qRT-PCR analysis. The results showed that ERRβ depletion is sufficient to abolish the p21^Waf1/Cip1^ upregulation by MLN4924 treatment in both MCF7 and MDA-MB-231 cell lines at the mRNA level (Fig. 5b, c, respectively), suggesting that ERRβ is responsible for the upregulation of p21^Waf1/Cip1^ expression at the transcriptional level by MLN4924 in both ER-positive and -negative breast cancer cells. We, next, performed luciferase reporter assay using a putative promoter region of the *p21*^*Waf1/Cip1*^ gene cloned upstream of the pGL2 luciferase reporter in MCF7 cells. The promoter assays again revealed ectopic expression of ERRβ causes an upregulation of the luciferase activity driven by the *p21*^*Waf1/Cip1*^ promoter (Fig. 5d). To confirm further that ERRβ is directly involved with the induction of p21^Waf1/Cip1^ transcription at the promoter level, we performed chromatin immunoprecipitation assay using a specific ERRβ antibody and showed ERRβ is recruited to the promoter region of the endogenous *p21*^*Waf1/Cip1*^ gene in MCF7 cells (Fig. 5e; left panel). Moreover, the binding of ERRβ to the promoter region of *p21*^*Waf1/Cip1*^ was also increased significantly after treatment with MLN4924 as revealed by ChIP-qPCR (Fig. 5e; right panel). Previous studies have shown that the transcription co-activator p300 is involved in the transcriptional function of ERβ and ERRβ^25–27^. To further confirm that ERRβ is involved in the regulation of *p21*^*Waf1/Cip1*^ transcription and explore the mechanism involved, we investigated whether p300 interacts with ERRβ and whether ERRβ is recruited to the *p21*^*Waf1/Cip1*^ gene promoter in the presence of MLN4924 in MCF7 cells. Co-immunoprecipitation experiments showed that the co-activator p300 bound to ERRβ, but the ERRβ levels did not increase substantially with MLN4924 treatment, indicating p300 levels are limiting in these cells (Fig. 5f). Notably, the molecular weight of p300-bound ERRβ species was higher than the majority of the unbound ERRβ (Fig. 5f), suggesting that the p300-bound ERRβ is post-translationally modified. ChIP-qPCR analysis showed that p300 was recruited directly to the *p21*^*Waf1/Cip1*^ promoter region (Fig. 5g; left panel). Notably, the binding of p300 was enhanced after MLN4924 treatment, suggesting that ERRβ recruits p300 to the *p21*^*Waf1/Cip1*^ gene promoter to augment its expression (Fig. 5g; right panel). Together these data suggested that MLN4924 induces ERRβ accumulation and that ERRβ recruits the transcription co-activator p300 to promote *p21*^*Waf1/Cip1*^ gene expression to restrict breast cancer growth.

### MLN4924 inhibits ERRβ-mediated breast cancer cell migration

**Fig. 6:**
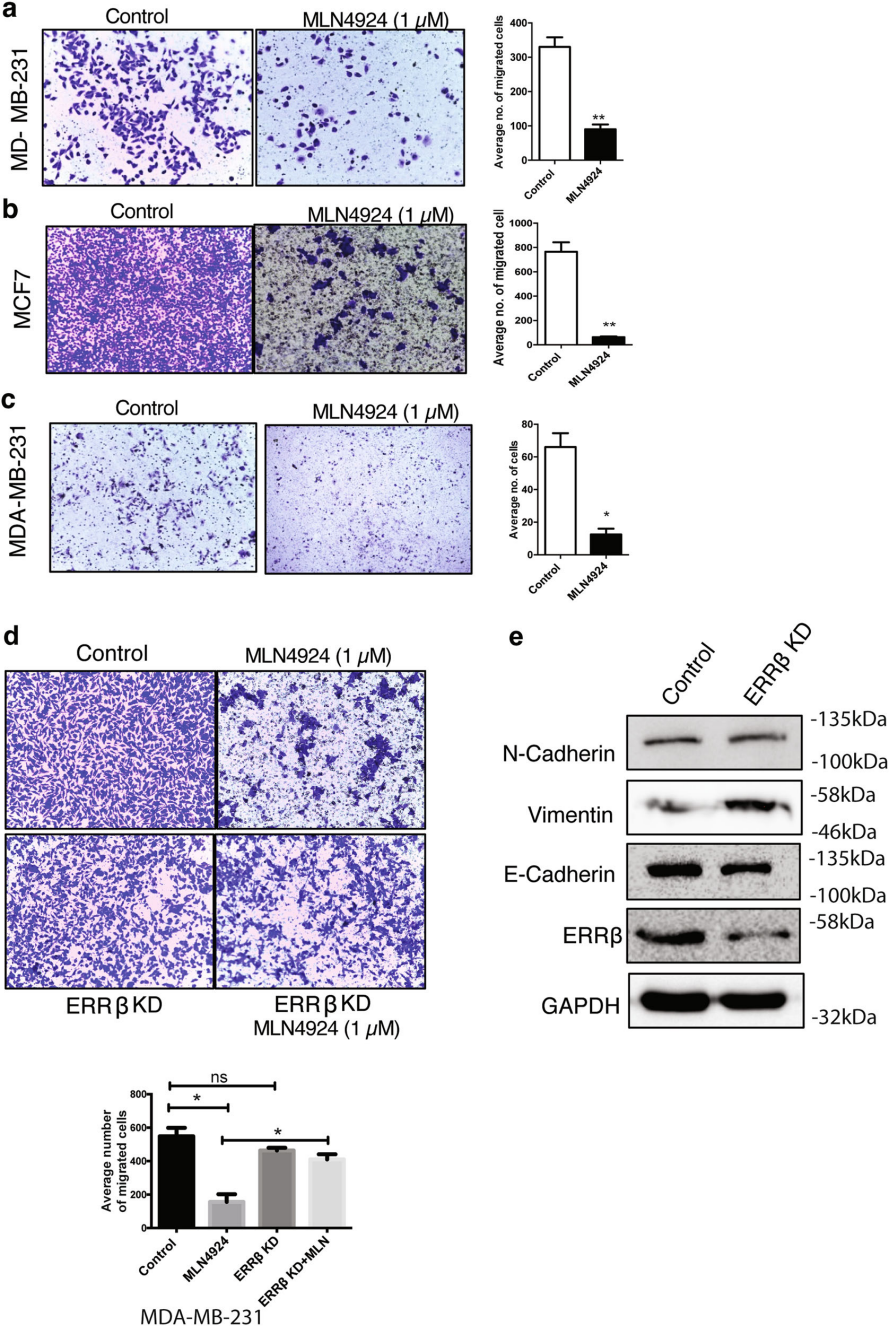
MLN4924 inhibits ERRβ-mediated breast cancer cell invasion and migration. **a** MDA-MB-231 and **b** MCF7 cells were treated with 1 µM MLN4924 for 24 h prior to Transwell-migration analysis. Graphical representation determined the average number of migrated cells. (*N* = 3). The data represent the means ± SD. (One-way ANOVA test; Significant: **P < 0.01, significant). **c** MDA-MB-231 cells were treated with 1 µM MLN4924 for 24 h prior to invasion analysis. Graphical representation determined the average number of cells. (*N* = 3). The data represent the means ± SD. (One-way ANOVA test; Significant: *P < 0.05, significant) **d** MDA-MB-231 cells were untreated, treated with 1 µM MLN4924 and/or ERRβ shRNA prior to invasion analysis. Graphical representation determined the average number of cells. (*N* = 3). The data represent the means ± SD. (One-way ANOVA test; Significant: *P < 0.05, significant; ns non-significant). Size bar:100 µm. **e** MDA-MB-231 cells were untreated, treated with 1 µM MLN4924 and/or ERRβ shRNA prior to western blot analysis for N-Cadherin, E-Cadherin, Vimentin, ERRβ and GAPDH expression. Representative blots are shown.

Cancer invasion and migration are prerequisite steps for metastasis^28,29^. To explore whether MLN4924 inhibits breast cancer cell migration, transwell-migration assays were performed with 5% serum as a chemoattractant on untreated and 1.0 μM MLN4924-treated MCF7 and MDA-MB-231 breast cancer cells. Consistent with our findings, MLN4924 significantly reduced cell migration after 24 h of MLN4924 treatment in both MCF7 and MDA-MB-231 cell lines (Fig. 6a, b). Next, we performed matrigel-coated transwell invasion assay to study the effect of MLN4924 on MDA-MB-231 cell invasion. Similar to cell migration, invasion was significantly reduced after MLN4924 treatment (1.0 μM) (Fig. 6c), suggesting further that MLN4924 has an anti-metastatic role in breast cancer cells. Next, we studied the effects of ERRβ knockdown on the clonogenicity of MLN4924-treated MDA-MB-231 cells. The results demonstrated that ERRβ silencing could partially reverse the effect of MLN4924 on migration (Fig. 6d). To confirm that further, the expression of E-Cadherin, N-Cadherin, and Vimentin were studied after ERRβ knockdown and the results showing the upregulation of N-Cadherin and Vimentin and the downregulation of E-Cadherin, were consistent with a switch to epithelial-to-mesenchymal transition (EMT) phenotype after ERRβ knockdown (Fig. 6e). Cell cycle analysis by FACS following propidium iodide staining also revealed an increase in population of cells at G2/M phase of the cell cycle in both MCF-7 and MDA-MB-231 cells after MLN4924 but the significance of this is not clear at this point (Supplementary Fig. S5).

**Fig. 7:**
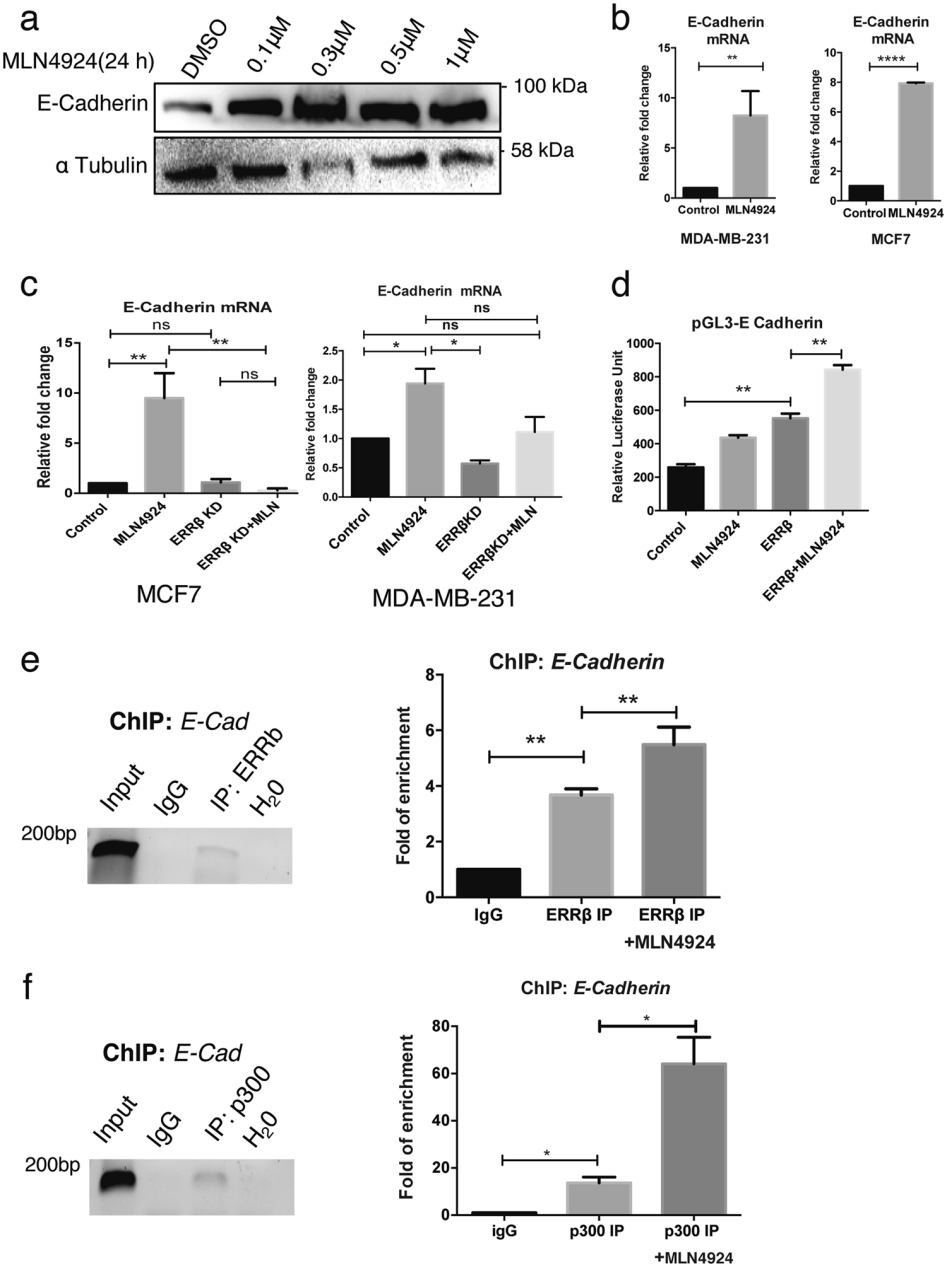
MLN4924 promotes ERRβ-mediated E-Cadherin expression. **a** MDA-MB-231 were treated with varying concentrations (0.1, 0.3, 0.5 and 1.0 µM) of MLN4924 for 24 h prior to western blot analysis. α-Tubulin was used as a loading control. (*N* = 3). **b** MDA-MB-231 (left panel) and MCF7 (right panel) cells were treated with 1 µM MLN4924 for 24 h prior to qRT-PCR analysis. (*N* = 3). The data represent the means ± SD. (Two-tailed *t* test; Significant: **P < 0.01; ****p < 0.0001, significant). **c** MDA-MB-231 (right) and MCF7 (left) cells were transfected with ERRβ shRNA and relative mRNA level of E-Cadherin were analysed after 1 µM MLN4924 treatment for 48 h. (*N* = 3). The data represent the means ± SD. (two-tailed *t* test; Significant: *P < 0.05; **P < 0.01, significant) **d** MCF7 cells were transfected with E-Cadherin promoter cloned upstream of the pGL3 luciferase reporter and ERRβ shRNA, with and without 1.0 µM MLN4924 treatment. Graphical representation showed relative luciferase unit of the Renilla luciferase reporter activity. (*N* = 3). The data represent the means ± SD (One-way ANOVA test; Significant: **P < 0.01, significant)**. e** Chromatin Immunoprecipitation was performed with the ERRβ antibody on the promoter region of E-Cadherin and analysed with electrophoresis (left; image colour invested) and qRT-PCR (right). Anti-IgG antibody was used as a negative control. (*N* = 3). **f** Chromatin immunoprecipitation was performed with a p300 antibody on the promoter region of endogenous E-Cadherin and analysed with electrophoresis (left; image colour invested) and qRT-PCR (right). Anti-IgG antibody was used as a negative control. (*N* = 3). The data represent the means ± SD. (two-tailed *t* test; Significant: *P < 0.05, significant).

To explore the potential underlying mechanism by which MLN4924 exerts its anti-metastatic function, we examined the expression and the regulation of E-Cadherin, an established negative modulator of EMT, a process important for cancer metastasis^29^. To this end, MDA-MB-231 cells were treated with 1 μM MLN4924 for 24 h. Western blot analysis showed that MLN4924 increased the expression of E-Cadherin in MDA-MB-231 cells in a dose-dependent manner (Fig. 7a). Next, we studied the effect of MLN4924 on E-Cadherin expression at the transcript level by qRT-PCR and found that MLN4924 dramatically increased E-Cadherin expression at the mRNA level in both MCF7 and MDA-MB-231 breast cancer cell lines (Fig. 7b). These results suggested that MLN4924 promotes E-Cadherin gene transcription and thus, increases E-Cadherin expression at least partially to limit breast cancer cell migration and metastasis.

To study the involvement of ERRβ in the MLN4924-mediated E-Cadherin upregulation, we performed qRT-PCR on MLN4924-treated MDA-MB-231 and MCF7 breast cancer cells with and without ERRβ knockdown and found that the knockdown of ERRβ significantly reduced E-cadherin mRNA levels even after MLN4924 treatment (Fig. 7c). This suggested that ERRβ is required for the MLN4924-induced E-Cadherin expression.

To confirm that the E-cadherin is transcriptional regulated by ERRβ, we performed luciferase reporter assay with a putative promoter region of E-cadherin cloned upstream of the luciferase reporter in MDA-MB-231 cells. The results showed that MLN4924 elevated the promoter activity of *E-Cadherin* and that overexpression of ERRβ also induced the *E-Cadherin* promoter activity (Fig. 7d). Moreover, treatment with MLN4924 increased further the effect of ERRβ overexpression on *E-Cadherin* promoter activity (Fig. 7d). Next, we sought to confirm the direct regulation of *E-Cadherin* gene expression by ERRβ using ChIP-qPCR (Fig. 7e) and found that ERRβ bound to the promoter region of E-Cadherin. Consistent with our promoter-reporter assay findings, ChIP analysis also demonstrated that MLN4924 treatment induced a further increase in the binding of ERRβ to the *E-cadherin* promoter (Fig. 7e).

As E-cadherin has been shown to be essential for suppressing cell migration and invasion, our data, therefore, suggested that the MLN4924-induced ERRβ accumulation directly inhibits cell migration and invasion at least partly through an upregulation of E-Cadherin at the transcriptional level. Previously, we showed that the co-activator p300 is recruited by ERRβ to the promoter region of *p21*^*Cip1*^ in MCF7 cells. To identify whether p300 is also involved in the upregulation of E-Cadherin expression, we performed ChIP-qPCR analysis and found that ERRβ can also promote the recruitment of p300 to the endogenous *E-cadherin* promoter in MDA-MB-231 cells (Fig. 7f). Collectively, our data suggested that MLN4924 promotes ERRβ expression and thereby, augments the recruitment of p300 to the promoter of *E-cadherin*, culminating in the transcriptional upregulation of E-Cadherin expression to restrict breast cancer migration.

## Discussion

ERRβ is an orphan nuclear receptor whose regulation is vastly unknown^30^. Here, for the first time, we reported that ERRβ is downregulated primarily at the protein level in breast cancer. In this study, we also uncovered that ERRβ degradation is mediated by the ubiquitin–proteasome pathway^31^. In mammalian cells, an estimated 500–1000 E3 ubiquitin ligases are responsible for targeting different proteins for proteasomal degradation^32^. The RING-finger E3 ligases are the largest family which contains ligases, such as the anaphase-promoting complex and SCF complex (Skp1–Cullin–F-box protein complex)^33^. We demonstrated an interaction of ERRβ with the Cullin1 subunit, suggesting that ERRβ is a substrate of SCF complex, which is involved in the downregulation of ERRβ in breast cancer. Previous studies have shown that NEDDylation of the Cullin subunits of the SCF complex enhances its activity^34^. We examined the components of the NEDDylation pathway in breast cancer tissue microarrays and different breast cancer cell lines and found that the components of NEDDylation cascade is overexpressed and the pathway hyperactivated in breast cancer. Consistent with our findings, NEDDylation is positively correlated with poor prognosis in different cancers^7,35,36^.

MLN4924 is a small molecule inhibitor of NAE which is a dimer of UBA3 and APP-BP1^22^. It has recently been reported that tumour regression properties of MLN4924 in ERα-positive breast cancer is dependent on the downregulation of ERα expression and its transcription activity^23^. In this study, we found that the MLN4924 inhibits Neddylation to block ERRβ ubiquitination and degradation, resulting in an accumulation of ERRβ in both ERα-positive and triple-negative breast cancer cells. We also showed that MLN4924 upregulates the expression of downstream ERRβ-target genes, including *p21*^*Waf1/Cip1*^ and *E-cadherin*, which have a role in restricting cancer cell proliferation, clonogenicity and migration^37^. Specifically, we showed that Neddylation inhibition by MLN4924 causes an increase in ERRβ and a decrease in the proliferative potential and clonogenicity of breast cancer cells. We also confirmed that ERRβ and the Neddylation inhibitor MLN4924 limit the proliferation and clonogenicity of breast cancer cells by up-regulating the expression of p21^Waf1/Cip1^. In particular, we showed that ERRβ binds directly the promoter region of *p21*^*Waf1/Cip1*^ and recruit its co-activator p300 to promote the transcription of *p21*^*Waf1/Cip1*^. MLN4924 has been demonstrated to possess anti-migratory properties in breast cancer^38^. In agreement, we have previously reported that ERRβ can restrict cancer cell migration through up-regulating the expression of E-cadherin^39^. As for p21^Waf1/Cip1^, we showed here that ERRβ binds directly to the promoter region of E-cadherin and enlist the co-transcription factor p300 to promote its transcription.

Previous proteomic analyses using MLN4924 and recombinant NEDD8 proteins have identified many direct and indirect Neddylation-targets important for cancer cell tumorigenesis, proliferation, migration and drug resistance^40–42^; however our data showed that ERRβ is a key mediator of the anti-proliferative and anti-migratory function of MLN4924 as ERRβ silencing can overcome its ability to induce the expression of key anti-proliferative and anti-migratory genes, such as *p21*^*Waf1/Cip1*^ and *E-cadherin*.

ERRβ has been shown to be a tumour suppressor and is frequently downregulated in breast cancer^30^. Collectively, our work uncovered a critical mechanism of ERRβ downregulation in breast cancer. Overactivation of the NEDDylation pathway is common in breast cancer and can induce ERRβ degradation through ubiquitination, leading to breast cancer progression. The Neddylation inhibitor MLN4924 restores ERRβ protein expression in breast cancer cells and can facilitate ERRβ-mediated cancer regression. Although MLN4924 has been reported to be an activator of the oncogenic AKT pathway, which is responsible for tumour progression and drug resistance, in HER2-positive and triple-negative breast cancer cell lines^38^, our data showed that the NEDDylation inhibitor can effectively repress ER-positive and triple-negative breast cancer cell proliferation, clonogenicity and cell migration. Consistent with our results, previous studies have demonstrated a role for the NEDDylation in breast cancer development and that the NEDDylation inhibitor MLN4924 has anti-breast cancer activity alone and in combination therapy^38,42,43^. Our findings on ERRβ regulation, mechanism of action and function can have a critical impact on the therapeutic intervention for breast cancer.

## Supplementary information

Supplementary Figure S1

Supplementary Figure S2

Supplementary Figure S3

Supplementary Figure S4

Supplementary Figure S5

Supplementary Figure Legends

Supplementary Materials and Methods
