# Supplementary figures and images for: NEDDylation negatively regulates ERRβ expression to promote breast cancer tumorigenesis and progression
Source: Cell Death Dis. 2020 Aug 24;11(8):703. doi: 10.1038/s41419-020-02838-7 (PMC7445179; doi:10.1038/s41419-020-02838-7)

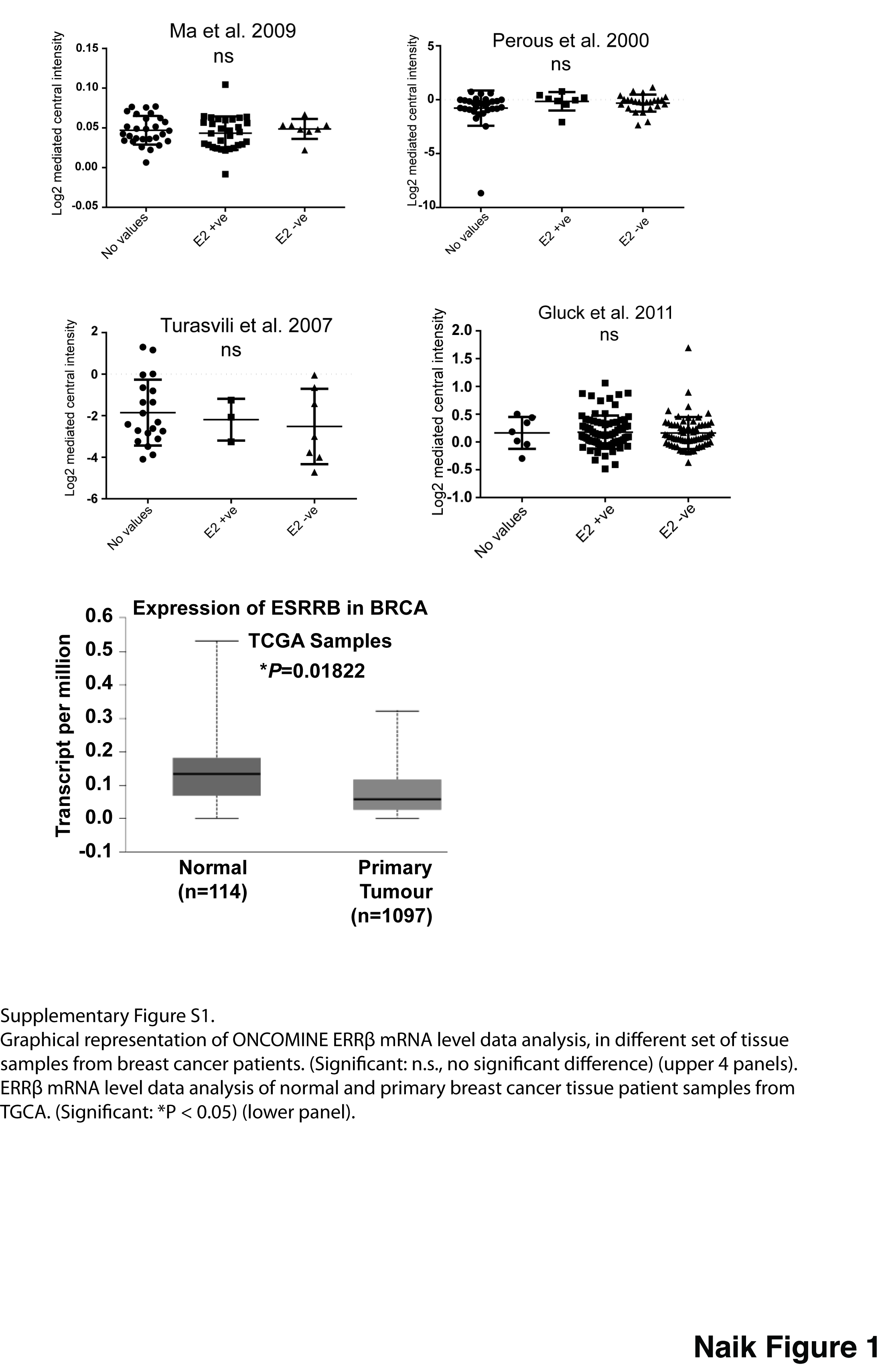

Supplement: Supplementary file 1 — Supplementary Figure S1 [file 41419_2020_2838_MOESM1_ESM.tif]

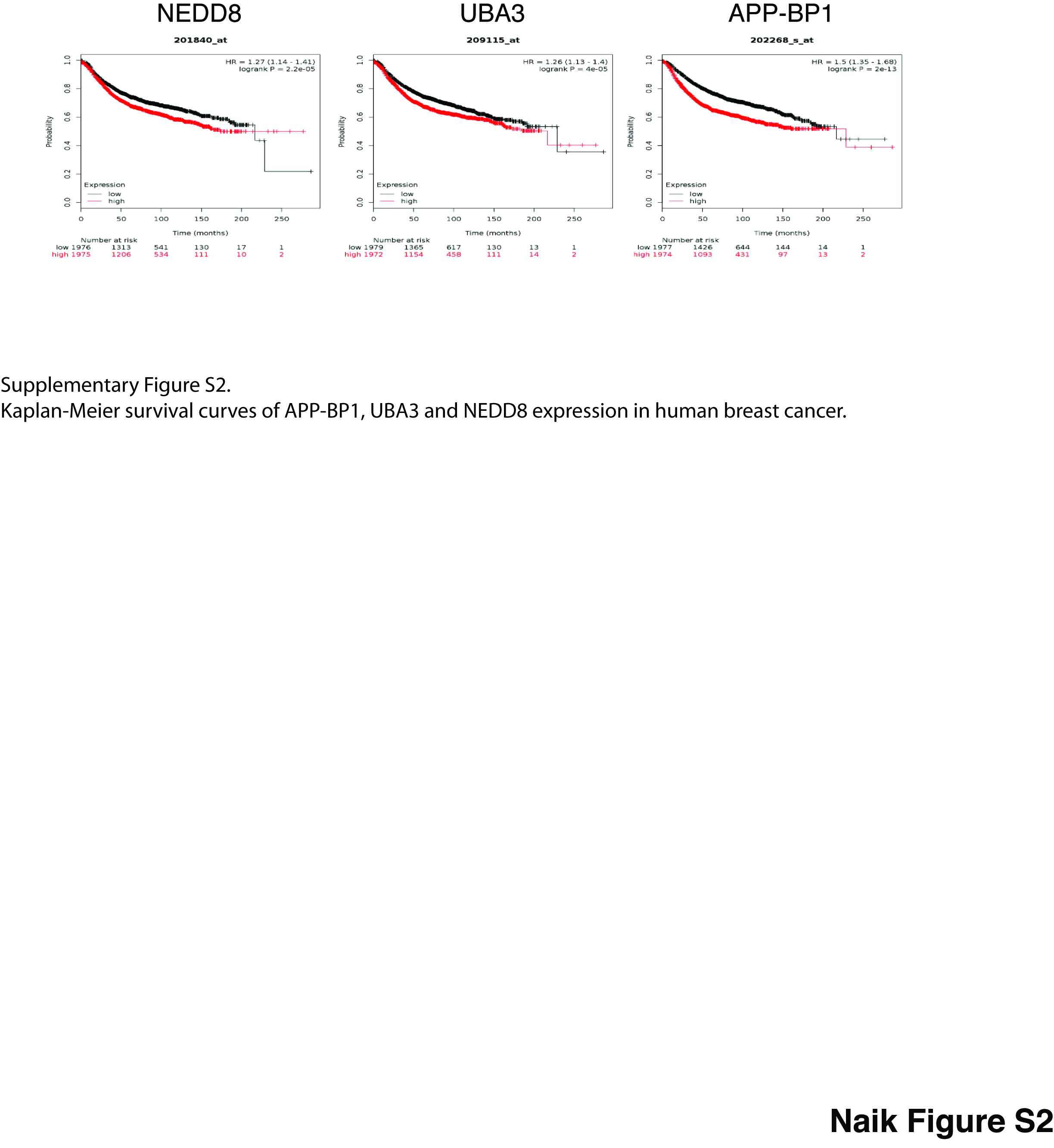

Supplement: Supplementary file 2 — Supplementary Figure S2 [file 41419_2020_2838_MOESM2_ESM.tif]

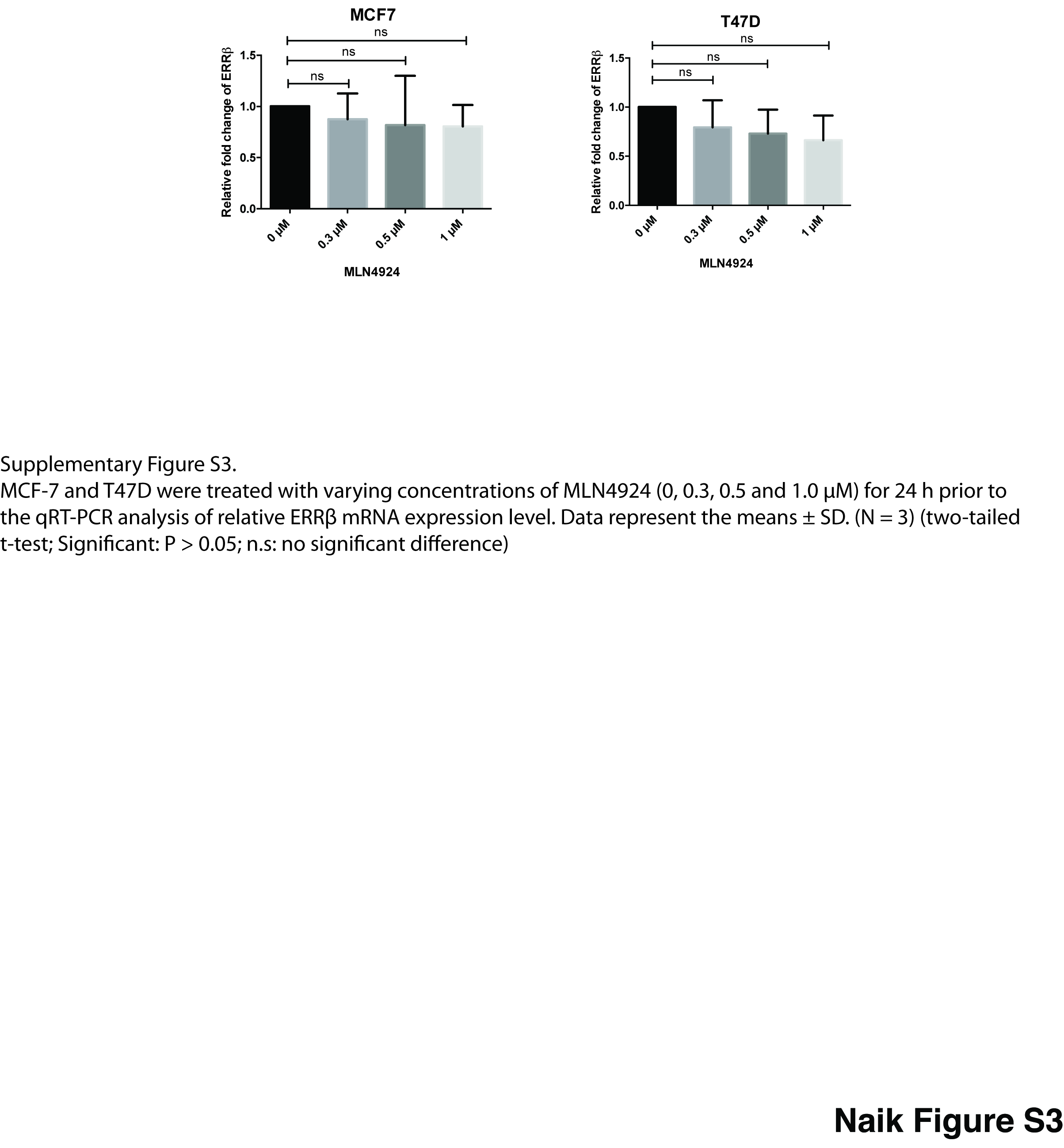

Supplement: Supplementary file 3 — Supplementary Figure S3 [file 41419_2020_2838_MOESM3_ESM.tif]

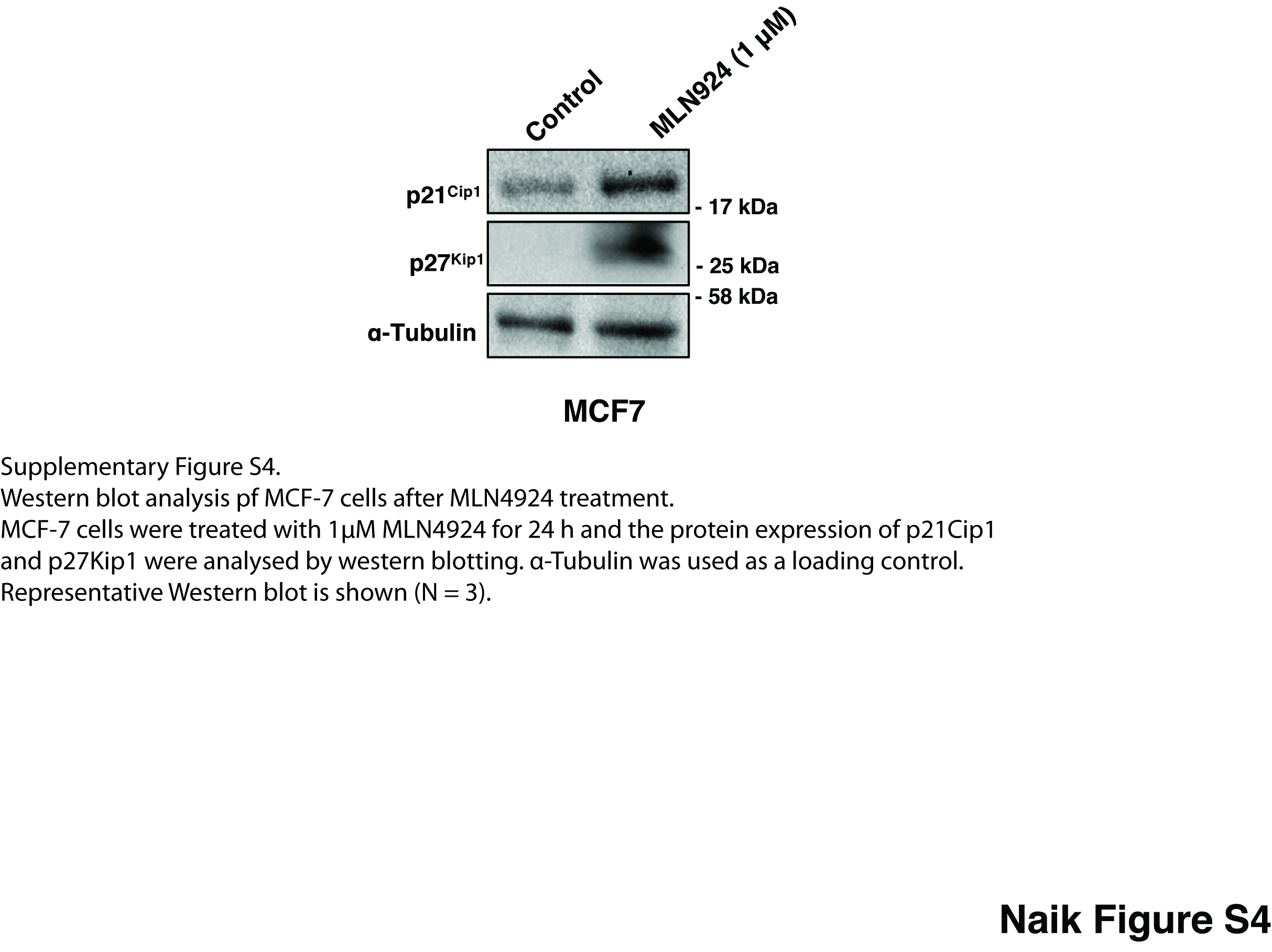

Supplement: Supplementary file 4 — Supplementary Figure S4 [file 41419_2020_2838_MOESM4_ESM.tif]

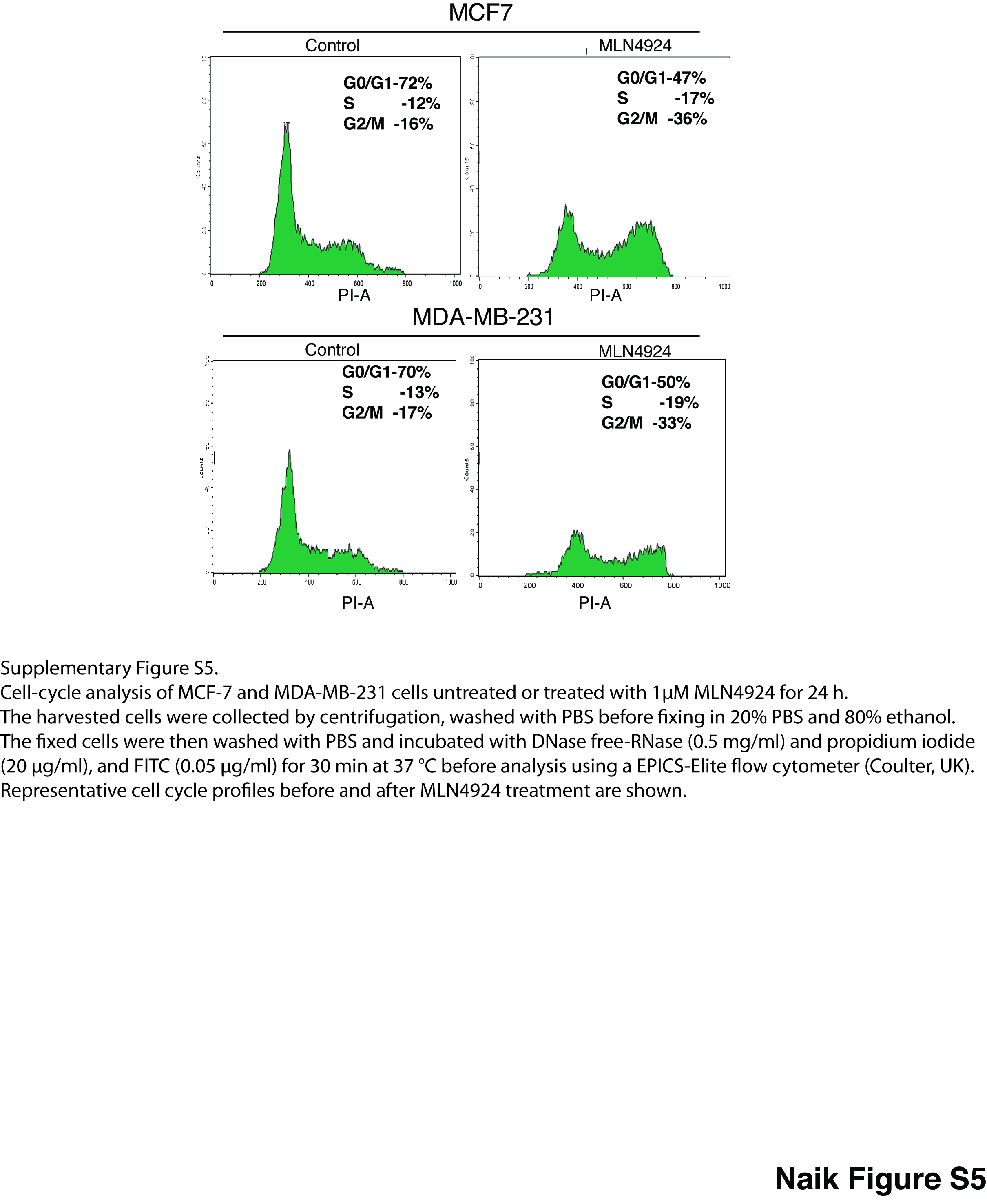

Supplement: Supplementary file 5 — Supplementary Figure S5 [file 41419_2020_2838_MOESM5_ESM.tif]
