# Supplementary material for: NEDDylation negatively regulates ERRβ expression to promote breast cancer tumorigenesis and progression
Source: Cell Death Dis. 2020 Aug 24;11(8):703. doi: 10.1038/s41419-020-02838-7 (PMC7445179; doi:10.1038/s41419-020-02838-7)
Supplement: Supplementary file 6 — Supplementary Figure Legends [file 41419_2020_2838_MOESM6_ESM.docx]

**Supplementary Figure Legends**

Supplementary Figure S1.

Graphical representation of ONCOMINE ERRβ mRNA level data analysis, in different set of tissue samples from breast cancer patients. (Significant: n.s., no significant difference) (upper 4 panels). ERRβ mRNA level data analysis of normal and primary breast cancer tissue patient samples from TGCA. (Significant: *P < 0.05) (lower panel).

Supplementary Figure S2.

Kaplan-Meier survival curves of APP-BP1, UBA3 and NEDD8 expression in human breast cancer.

Supplementary Figure S3.

MCF-7 and T47D were treated with varying concentrations of MLN4924 (0, 0.3, 0.5 and 1.0 µM) for 24 h prior to the qRT-PCR analysis of relative ERRβ mRNA expression level. Data represent the means ± SD. (N = 3) (two-tailed t-test; Significant: P > 0.05; n.s: no significant difference)

Supplementary Figure S4.

Western blot analysis pf MCF-7 cells after MLN4924 treatment.

MCF-7 cells were treated with 1µM MLN4924 for 24 h and the protein expression of p21^Cip1^ and p27^Kip1^ were analysed by western blotting. α-Tubulin was used as a loading control. Representative Western blot is shown (N = 3).

Supplementary Figure S5.

Cell-cycle analysis of MCF-7 and MDA-MB-231 cells untreated or treated with 1µM MLN4924 for 24 h. The harvested cells were collected by centrifugation, washed with PBS before fixing in 20% PBS and 80% ethanol. The fixed cells were then washed with PBS and incubated with DNase free-RNase (0.5 mg/ml) and propidium iodide (20 μg/ml), and FITC (0.05 μg/ml) for 30 min at 37 °C before analysis using a EPICS-Elite flow cytometer (Coulter, UK). Representative cell cycle profiles before and after MLN4924 treatment are shown.
