# Supplementary material for: NEDDylation negatively regulates ERRβ expression to promote breast cancer tumorigenesis and progression
Source: Cell Death Dis. 2020 Aug 24;11(8):703. doi: 10.1038/s41419-020-02838-7 (PMC7445179; doi:10.1038/s41419-020-02838-7)
Supplement: Supplementary file 7 — Supplementary Materials and Methods [file 41419_2020_2838_MOESM7_ESM.docx]

**Supplementary Materials and Methods**

**Cell culture**

Breast cancer cell lines MCF7, T47D and MDA-MB-231 were purchased from National Centre for Cell Sciences (NCCS, Pune, India). MCF7 cells were cultured in Dulbecco’s Modified Eagle’s Medium (DMEM), whereas T47D and MDA-MB-231 cells in RPMI supplemented with 10% fetal bovine serum (FBS) and penicillin-streptomycin (MP Biomedicals, Bengaluru, India) at 37 °C, 5% CO_2_ and 95% humidity. MCF10A, a kind gift from Dr. Annapoorni Rangarajan (IISC, Bangalore, India), was maintained in DMEM F12 containing horse serum supplemented with hydrocortisone, EGF, insulin, cholera toxin and penicillin-streptomycin at 37 °C, 5% CO2 and 95% humidity. The cells were grown until 70-80% confluence and subcultured using Trypsin-EDTA.

*Cell viability assay*

The effect of MLN4924 on viability of cells was examined by 3-(4,5-Dimethylthiazol-2-yl)-2,5 Diphenyltetrazolium Bromide (MTT) assay. MCF7, MDA-MB-231 cells were seeded at a density of 3 × 10^3^ cells per well in 96-well plates. The cells were treated with different concentrations of MLN4924 (0.01, 0.02, 0.03, 0.06, 0.13, 0.25, 0.5, 1, 2, 4 µM) and incubated for 24 hours. After appropriate incubation time, 10 μL of MTT (MP Biomedical) (5 mg/mL in PBS) was added into each well and incubated at 37 °C, 5% CO_2_ atmospheric condition for another 3 hours. After incubation, the medium was removed and 100 μL of DMSO was added to dissolve thus formed formazan crystals. The solubilized crystals were then quantified by scanning the plates at 570 nm using Varioskan™ Flash Multimode Reader (Thermo Fisher Scientific, Bangalore, India). Three independent sets of experiments were performed to evaluate the effect of MLN4924. The percent viability was calculated by the formula: % viability = A/A0 X 100 where A0 and A are the absorbance of vehicle control and MLN4924 treated cells respectively.

*Colony forming assay*

For colony forming assay, 0.6 X 10^3^ of MCF-7 and MDA-MB-231 cells were seeded in triplicates in 60mm-plates (Corning, Pune, India) and after 24 h of cell attachment, the cells were treated with 1 μM of MLN4924. The plates were under incubation at 37 °C, 5% CO2 to allow the growth of colonies (~50 cells per colony). During long-term incubation, fresh complete growth medium with 1 μM of MLN4924 was replaced every day. The cells were washed twice with 1X PBS (137 mM NaCl, 2.7 mM KCl, 10 mM Na2HPO4 and 2 mM KH2PO4), fixed with 10% (v/v) formalin and then stained with 0.01% (w/v) crystal violet solution. The excess stain was removed by washing with 1X PBS. The plate was air-dried, and representative images were captured using Gel Doc™ XR + Imager (Bio-Rad).

*Transwell migration and invasion assays*

Transwell-migration assay was performed following manufacturer’s protocol (BD Falcon, Bhubaneswar, India). Appropriately MLN4924 (1 μM, 24 h) treated MCF-7 and MDA-MB-231 cells were seeded at a density of 2.5 × 10^4^ cells in the upper chamber of 12-well transwell system in 500 μL of serum and phenol red free DMEM. Medium supplemented with 5% serum was used as chemo-attractant in the lower chamber. After 24 h, the cells on both sides of the membrane were fixed with 10% formalin and stained with 0.01% crystal violet stain. The cells were scrubbed on the seeded side to quantify the percent of migrated cells only. The membrane was then washed with 1XPBS and the cells attracted towards the serum were visualized under light microscope and pictured (10X) under different field views. The number of migrated cells in control and MLN4924 treatment in 10 different fields were calculated using ImageJ software and the average value was represented in the graph. For invasion assay, the transwell migration chamber was coated with matrigel (2 mg/ml) (BD Biosciences, Bhubaneswar, India). The cells present towards the lower side of the chamber were considered as invaded cells and were fixed and stained with crystal violet similar to migration assay.

*Tissue microarray*

Breast cancer tissue microarray slides (Cat No. BR 246a) were purchased from US Biomax (Rockville, MD, USA). The slides were stained by anti-APP-BP1 antibody at 1:50 dilution (PAC219Hu01 Cloud-Clone Corp. USA) and were further processed using ABC system (Vector Laboratories, Bulingame, CA, USA) as described previously {Lopez, 2016 #19}. The images were captured under Leica microscope (Wetzlar, Germany) using LAS EZ software version 2.1.0. The slides were examined, and score was done by an experienced pathologist. The intensity score was calculated based on staining for ERRβ and was assigned from 0 to 3 (0 indicates no staining; 1+ weakly stained; 2+ moderately stained and 3+ strongly stained positively). The percentage of positively stained cells were scored as follows: 0, no positive staining; 1+, 1–25% positively stained cells; 2+, 26–50% positively stained cells; 3+, 51–70% positively stained cells and 4+, > 70% positively stained cells. The composite score was calculated using both intensity score and the percentage of positive cells as it is a product of both scores. The composite score range was given from 0 to 12. The samples scored < 3 were considered as low categorized; 3–5 moderately categorized; ≥ 6 highly categorized. The graph was plotted using composite scores using GraphPad Prism version 6.01 (San Diego, CA, USA).

*Western blot analysis*

For western blot, whole-cell lysate of cells were prepared using RIPA buffer [20 mM Tris-HCl (pH 7.5), 150 mM NaCl, 1 mM Na_2_EDTA, 1 mM EGTA, 1% NP-40, 1% sodium deoxycholate, 2.5 mM sodium pyrophosphate, 1 mM β-glycerophosphate, 1 mM Na3VO4 and 1 μg/mL leupeptin] as previously described {Intuyod, 2018 #83}. The lysed samples were collected after centrifugation for 15 min at 12,000 x *g*, 4 °C. Equal amount (30 μg) of proteins were loaded after Bradford method of protein quantification. The samples were run in 10% SDS-PAGE gel, transferred on PVDF membrane (Millipore) and blocked with 5% (w/v) non-fat milk (Sigma, St Louis, MO, USA). Blots were then incubated with primary antibody overnight [ERRβ (1:5000)(Sc-68879) (Santa Cruz, CA, USA), NEDD8 (1:5000) (GTX54567) (Zeeland, Michigan, United States), App-Bp1 (1:5000) (PAC219Hu01 Cloud-Clone Corp. USA), UBA3 (1:5000) (HPA034873) (Sigma, St Louis, MO, USA), GAPDH (1:10000) (sc-365062) (Santa Cruz, CA, USA), p21^CIP1^ (1:5000) (CST-99323) (Europe, B.V), α-tubulin (1:1000) (T9026) (Sigma St Louis, MO, USA), E-Cadherin (1:5000) (CST-9782T) (Europe, B.V), p300 (1:5000) (P2859) (Sigma St Louis, MO, USA), Thereafter, 1 h with their respective HRP conjugated secondary antibody [anti-rabbit (1:5000, Sigma Aldrich) or anti-mouse (1:5000, Sigma Aldrich)], the blots were subjected to chemi-lumenescent detection reagent for visualization and the bands were detected by using Gel Doc™ XR + Imager. Densitometry analyses of the protein bands were calculated by using ImageJ software.

*Transfection and luciferase assays*

MCF7 cells were grown in 24 well-plates in phenol red free DMEM supplemented with 10% (v/v) charcoal treated fetal bovine serum, 24 h prior to estrogen (E2) treatment. Cells were transfected with, pEYFP C1-ERRβ, pGL2-P21, pGL3-E-Cadherin, and pRL-Renilla luciferase constructs (Promega Biotech, Bhubaneswar, India) in different combinations using jetPRIME-polyplus-transfection reagent (Polyplus transfection, New York, NY, USA) according to manufacturer’s protocol. Luciferase assay was performed using Dual luciferase assay detection kit (Promega) according to manufacturer’s protocol. Luciferase readings were obtained and were normalized with Renilla luciferase activity. The graph was plotted with normalized readings using GraphPad Prism software version 6.01.

*Co-immunoprecipitation*

Cells were washed with PBS pH 7.4 twice and lysed with NP40 buffer (50 mM Tris-Cl pH 8.0, 150 mM NaCl, 1% NP40). Lysates were precleared by the addition of 50 μl of agarose beads for 30 min. Total protein (600 μg) and 4 μg of antibody were used for each IP and rotated overnight in 4 °C. Beads (30 μg) were added to each IP and rotated for 2 h, followed by centrifugation at 500 x *g* for 3 min. Supernatants were removed, and pellets were washed four times with NP40 buffer. Complexes were eluted in SDS lysis buffer.

*Chromatin immunoprecipitation assay (ChIP)*

Chromatin immunoprecipitation was performed as performed as previously described with minor modifications. MCF7 and MDA-MB-231 cells were lysed in SDS lysis buffer (1% (w/v) SDS, 10 mM EDTA, 50 mM Tris-HCl (pH 8.1)) with protease inhibitor cocktail (Sigma-Aldrich) and were sonicated using Bioruptor ultrasonicator device (Diagenode S.A., Seraing, Belgium) at M2 amplitude strength. The sonicated samples were subjected to pre-clearing with protein A/G agarose beads (GE Healthcare Life Sciences). These pre-cleared samples were diluted with ChIP dilution buffer (0.01% (w/v) SDS, 1.1% (v/v) Triton X-100, 1.2 mM EDTA, 16.7 mM Tris-HCl (pH 8.1), 167 mM NaCl) and divided into two equal parts IgG and IP, 50 μl was taken as input and was stored at -80 °C. The IgG and IP were incubated with 1 μg of anti-IgG (Diagenode), anti-ERRβ (sc-68879, Santa Cruz) and anti-P300 (P2859 Sigma St Louis, MO, USA) antibodies respectively. The protein-antibody complex was extracted by incubating the samples with protein A/G agarose beads. The protein-antibody-bead complex was extracted, washed with series of different washing buffers i.e. Low salt buffer [0.1% (v/v) SDS, 2 mM EDTA, 1% (v/v) Triton X-100, 20 mM Tris-HCl (pH 8.1) and 150 mM NaCl], High salt buffer [0.1% (v/v) SDS, 1% (v/v) Triton X-100, 2 mM EDTA, 20 mM Tris-HCl (pH 8.1) and 500 mM NaCl], LiCl salt buffer [0.25 M LiCl, 1% (v/v) NP-40, 1% (w/v) deoxycholic acid (sodium salt), 1 mM EDTA and 10 mM Tris-HCl (pH 8.1)], 1X TE [10 mM Tris-HCl (pH 8.1) and 1 mM EDTA] and were eluted using elution buffer (1% (v/v) SDS, 0.1 M NaHCO3). The eluted samples and input were reverse crosslinked with 5 M NaCl for 6 h at 65 °C followed by incubation with 0.5 M EDTA, 1 M Tris-HCl (pH 6.5) and proteinase K at 45 °C for 1 h. ChIP elutes were purified using phenol/chloroform and ethanol precipitated. DNA samples were further used to perform PCR analyses to confirm the binding of ERRβ and p300 on *p21^Cip1^* and *E-Cadherin* promoter. The primer sequences used for ChIP-PCR were provided in **Table 1.**

| Human genes | | Primer sequences (5’-3’) |
| --- | --- | --- |
| E-Cadherin | F | TAGAGGGTCAC CGC GTCTAT |
|  | R | TCACAGGTGCTTTGCAGTTC |
| p21^Cip1^ | F | CACTGCTGACTTTGTCTCAAAAAAC |
|  | R | CTGCAGAAGCTGCCTAGGAAG |

**Table 1. List of specific primers for ChIP-PCR.**

| Human genes | | Primer sequences (5’-3’) |
| --- | --- | --- |
| ERRβ | F | CTATGACGACAAGCTGGTGT |
|  | R | CCTCGATGTACATGGAATCG |
| E-Cadherin | F | GCCTCCTGAAAAGAGAGTGGAAG |
|  | R | TGGCAGTGTCTCTCCAAATCCG |
| p21Cip1 | F | GAGGCCGGGATGAGTTGGGAGGAG |
|  | R | CAGCCGGCGTTTGGAGTGGTAGAA |
| GAPDH | F | AAGATCATCAGCAATGCCTC |
|  | R | CTCTTCCTCTTGTGCTCTTG |

# Table 2. List of specific primers for qRT-PCR.
